# JAK-STAT pathway heterogeneity governs immunotherapy response in breast cancer

**DOI:** 10.7554/eLife.110635

**Published:** 2026-10-13

**Authors:** Jianbo Zhou, Heng Zhang, Hailin Tang, Lei Yu, Fu Peng

**Affiliations:** 1 West China School of Pharmacy, Department of Thoracic Surgery and Institute of Thoracic Oncology, Frontiers Science Center for Disease-Related Molecular Network, West China Hospital, Sichuan University Chengdu China; 2 State Key Laboratory of Oncology in South China, Guangdong Provincial Clinical Research Center for Cancer, Sun Yat-Sen University Cancer Center Guangzhou China; 3 Chongqing Hospital of Traditional Chinese Medicine Chongqing China; Institute of Radiation Medicine, Chinese Academy of Medical Sciences and Peking Union Medical College China; Shengjing Hospital of China Medical University China

**Keywords:** breast cancer, immunotherapy, JAK-STAT pathway, Human

## Abstract

The JAK-STAT pathway (JSP) is a well-known oncogenic cascade; however, recent clinical trials have detected JSP upregulation in breast cancer following anti-PD-1 immunotherapy. This paradoxical observation warrants further investigation into JSP’s intercellular heterogeneity, tumor dynamics, molecular mechanisms, and clinical implications for immunotherapy. JSP expression showed dynamic shifts during breast cancer progression, with higher levels in T cells and paracancerous epithelial cells. In human breast cancer tumor cells, elevated JSP is highly correlated with malignant phenotypes. JSP-high tumor cells overexpressed oncogenic pathways, while exhibiting increased immunosuppressive signaling via the MIF-CD74 signaling axis. In T cells, higher JSP levels were associated with enhanced cytotoxic activity, improved differentiation, and reduced exhaustion, reflecting robust anti-tumor immunity. Analysis of immunotherapy datasets revealed that higher JSP levels were associated with improved responses toward PD-1 inhibitors, particularly in triple-negative breast cancer (TNBC) patients, with JSP serving as a predictive biomarker for immunotherapy sensitivity. As a key JSP component, STAT4 exerts dual roles in breast cancer: it drives tumorigenesis in malignant cells, sustains breast epithelial cell proliferation, and bolsters T-cell anti-tumor functionality, while also acting as a highly accurate biomarker for predicting immunotherapy response. This indicates that JSP targeting demands a nuanced approach: broad inhibition may impair anti-tumor immunity, and optimized therapeutic strategies paired with precise biomarkers are critical to maximize JSP’s utility in breast cancer immunotherapy. Our findings highlight JSP’s functional heterogeneity in epithelial, tumor, and T cells, with high JSP activity correlating with enhanced immunotherapy efficacy in breast cancer.

## Introduction

Breast cancer is the most common cancer in women, accounting for 32% (321,910 cases) of new cancer diagnoses and 14% (42,140 cases) of cancer-related deaths among women in the United States, 2026 (Siegel et al., 2026). Globally, approximately 2.3 million new breast cancer cases and 670,000 breast cancer-related deaths are reported in 2022 (Kim et al., 2025). Breast cancer is commonly classified by immunohistochemistry based on the expression of estrogen receptor (ER), progesterone receptor (PR), and human epidermal growth factor receptor 2 (HER2). Using these markers, tumors are typically divided into four major subtypes—luminal A, luminal B, HER2-positive, and triple-negative breast cancer (TNBC). Clinically, this scheme is often simplified into three groups: luminal (ER and/or PR positive, HER2 negative), HER2-positive, and TNBC (ER, PR, and HER2 all negative) (Harbeck and Gnant, 2017). TNBC accounts for approximately 15% of all breast cancers, with a rising overall incidence in recent years and higher prevalence among young women (Kim et al., 2025). It can be further classified into distinct molecular subgroups, including basal-like (BL1 and BL2), claudin-low, mesenchymal-like (MES), luminal androgen receptor (LAR), and immunomodulatory (IM) types (Wu et al., 2021b; Ensenyat-Mendez et al., 2021). Among these, basal-like subtypes predominate, with BL1 and BL2 representing the largest proportions. An alternative molecular classification based on genomic biomarkers divides TNBC into four subtypes: LAR, IM, basal-like immune-suppressed, and MES (Fan et al., 2024). TNBC is characterized by aggressive behavior, a high risk of early relapse, and a propensity to present at advanced stages. Histologically, TNBCs are often poorly differentiated, highly proliferative, and heterogeneous, encompassing subsets with variable prognoses. Immunohistochemically, TNBC can be categorized as basal or non-basal: basal tumors typically express cytokeratins CK5/6 and EGFR, whereas non-basal tumors do not express CK5/6. Notably, approximately 80% of tumors associated with BRCA1/BRCA2 alterations fall into the TNBC category (Jie et al., 2025). Current standard treatments include surgery, radiotherapy, neoadjuvant chemotherapy, or/and immunotherapy with immune checkpoint inhibitors (ICIs) (e.g. anti-PD-1: pembrolizumab; anti-PD-L1: atezolizumab; anti-CTLA4: tremelimumab) (Xiong et al., 2024; Xiong et al., 2025). Although immunotherapy has reshaped breast cancer treatment patterns and improved patients’ survival benefits, heterogeneity in treatment responses and the lack of robust predictive biomarkers continue to limit its optimal clinical application (Li et al., 2023b; Debien et al., 2023; An et al., 2026).

The JAK-STAT (Janus kinase-signal transducer and activator of transcription) pathway is composed of cytokine receptors (e.g. IL-2, IL-6, IL-10, PDGF, epidermal growth factor [EGF]) (Hu et al., 2021), the JAK family (JAK1, JAK2, JAK3, and TYK2), the STAT family (STAT1-6, STAT5a, and STAT5b), and negative regulatory factors. These negative regulators, including the SOCS family, PIAS family, and protein tyrosine phosphatases, inhibit the activation of the JAK-STAT pathway (JSP) by suppressing the phosphorylation of JAK or/and STAT proteins (Liang et al., 2024), while PP2A and NPM-ALK maintain STAT3 phosphorylation (Seffens et al., 2019). In the canonical working model of JAK-STAT signaling, cytokine binding to its cognate receptor (e.g. gp130, EpoR, TNF-R1, IL-17R, IL-10R) triggers the phosphorylation and activation of JAKs. Activated JAKs then phosphorylate STATs, inducing their dimerization. The dimeric STATs translocate into the nucleus and regulate the transcription of target genes (Hu et al., 2021; Parveen et al., 2025). This pathway is regarded as one of the major abnormally activated oncogenic pathways in breast cancer, contributing to tumor proliferation, invasion, and metastasis (Xiong et al., 2024) and collaborating with TGF-β, MAPK, Notch, PI3K/AKT/mTOR, and NF-κB signaling pathways (Hu et al., 2021).

Interestingly, the JSP operates as a double-edged sword in tumor immunity, mediating both pro- and anti-tumor programs within the tumor microenvironment (TME) (Owen et al., 2019). On the pro-tumor side, signaling through STAT3 and STAT5 drives immunosuppressive networks in helper T cells, regulatory T cells, and myeloid-derived suppressor cells (MDSCs), with downstream cytokines such as IL-6 and IL-23 reinforcing tumor-promoting inflammation and immune evasion. Conversely, anti-tumor activity in the TME is largely orchestrated by STAT1 and STAT4. Th1-associated axes—notably IL-12/STAT4/IL-2 and IFN-γ/STAT1/IFN-γ—foster expansion and activation of CD8^+^ T cells and NK cells. In these cytotoxic lymphocytes, IL-12-STAT4 and IFN-α/β-STAT1 signaling induce granzyme B and IFN-γ production, initiating tumor cell killing (Watford et al., 2004). In dendritic cells (DCs), type I interferon signaling via STAT1 (and, in some contexts, STAT3) upregulates key effector mediators such as IL-12, TNF-α, and interferons, thereby enhancing antigen presentation and T-cell priming (Owen et al., 2019). Additionally, STAT3 maintains proliferation of naïve CD8^+^ T cells, contributes to the initial expansion of activated CD8^+^ T cells, and facilitates the differentiation from memory CD8^+^ T cells to effector CD8^+^ T cells through upregulating the key transcriptional factor Bcl-6 (Olson and Jameson, 2011).

**Figure 1.**
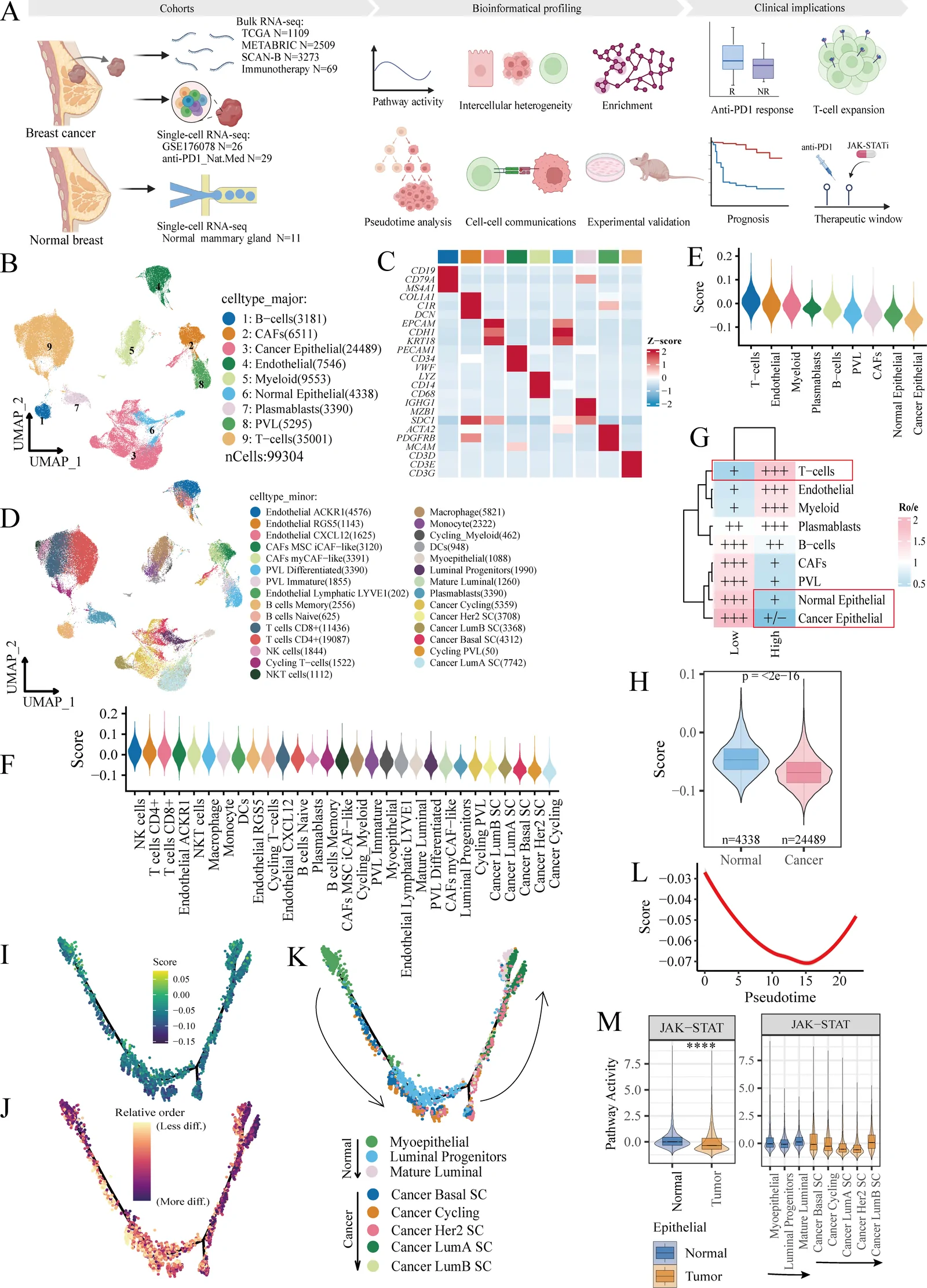
Heterogeneous expression pattern of JAK-STAT pathway in breast cancer. (**A**) Schematic illustration of the overall experimental and analytical workflow. (**B**) UMAP plot illustrating the major cell populations derived from the single-cell dataset GSE176078. (**C**) Core marker gene expressions of major cell populations. (**D**) UMAP plot depicting the minor cell subpopulations. (**E, F**) Distribution of JAK-STAT pathway scores across major and minor cell types. (**G**) Ro/e index for JAK-STAT pathway score. (**H**) JAK-STAT pathway scores in paracancerous epithelial cells versus cancerous epithelial cells. (**I**) Pseudotime trajectory analysis of epithelial cell states using Monocle2. (**J, K**) Pseudotime scores computed by Monocle2 and CytoTRACE2-derived cytotrace scores (in which higher scores indicate lower levels of differentiation). (**K**) Differentiation trajectories of epithelial cell subtypes. (**L**) Pseudotime dynamics of the JAK-STAT pathway scores. (**M**) Activity of the JAK-STAT pathway within epithelial cells.

Recent studies have observed activation of JSP following neoadjuvant immunotherapy (PD-1 inhibitor), and this activation was considered to contribute to treatment response and pathological complete response in breast cancer (Voorwerk et al., 2019; Zhang et al., 2025). The apparent paradox—that JAK-STAT signaling can drive tumorigenesis yet becomes hyperactivated following immunotherapy—prompted us to dissect its context-dependent roles within TME. We combined bulk RNA-seq with single-cell RNA-seq (scRNA-seq) from primary tumors and immunotherapy cohorts to map JAK-STAT dynamics during the transition from normal to malignant epithelium and to chart its heterogeneity across epithelial cells and T cells (Figure 1A). We find that JAK-STAT signaling is essential for epithelial differentiation and T-cell cytotoxicity; in immunotherapy-treated cohorts, the pathway activation is associated with enhanced T-cell-mediated cytotoxicity and predicts clinical response and T-cell expansion. Multi-omics profiling and experiments further identify STAT4 as a salient marker and mediator of JAK-STAT activity. Altogether, these results delineate distinct biological roles and molecular dependencies of the JAK-STAT axis in TME and argue that precise modulation of this pathway could augment immunotherapy efficacy while limiting harm to normal epithelial cells and T cells.

**Figure 1—figure supplement 1.**
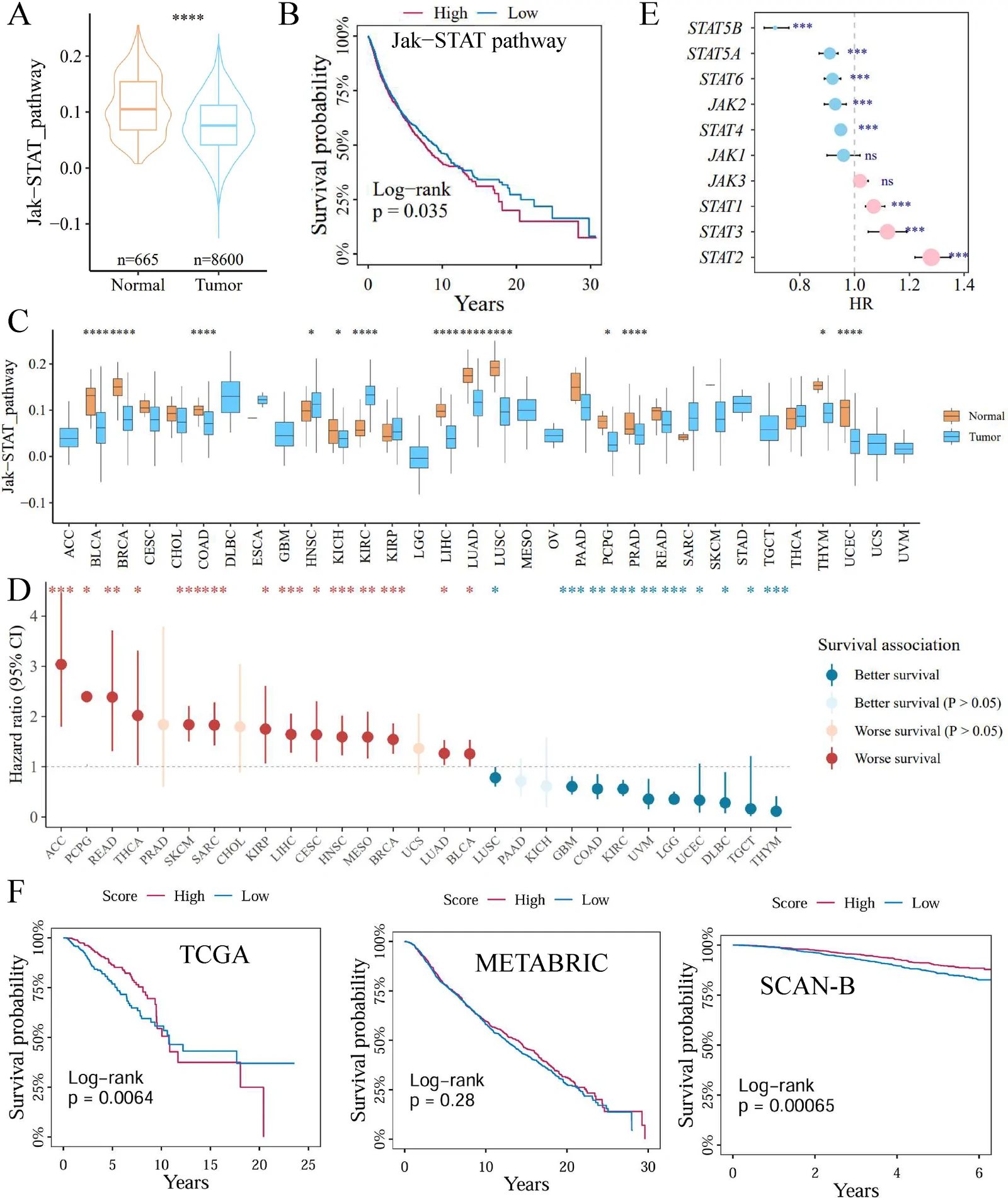
The JAK-STAT signaling pathway is abnormally expressed and prognosis-related in cancers. (**A**) Expression of JAK-STAT pathway (JSP) in adjacent non-tumor and tumor tissues across a pan-cancer cohort. (**B**) Survival curves based on JSP expression, stratified by median value. (**C**) Expression of JSP in adjacent non-tumor and tumor tissues within specific cancer types. (**D**) Univariate COX analysis across different cancer types. (**E**) Univariate COX analysis of core JSP components.

**Figure 1—figure supplement 2.**
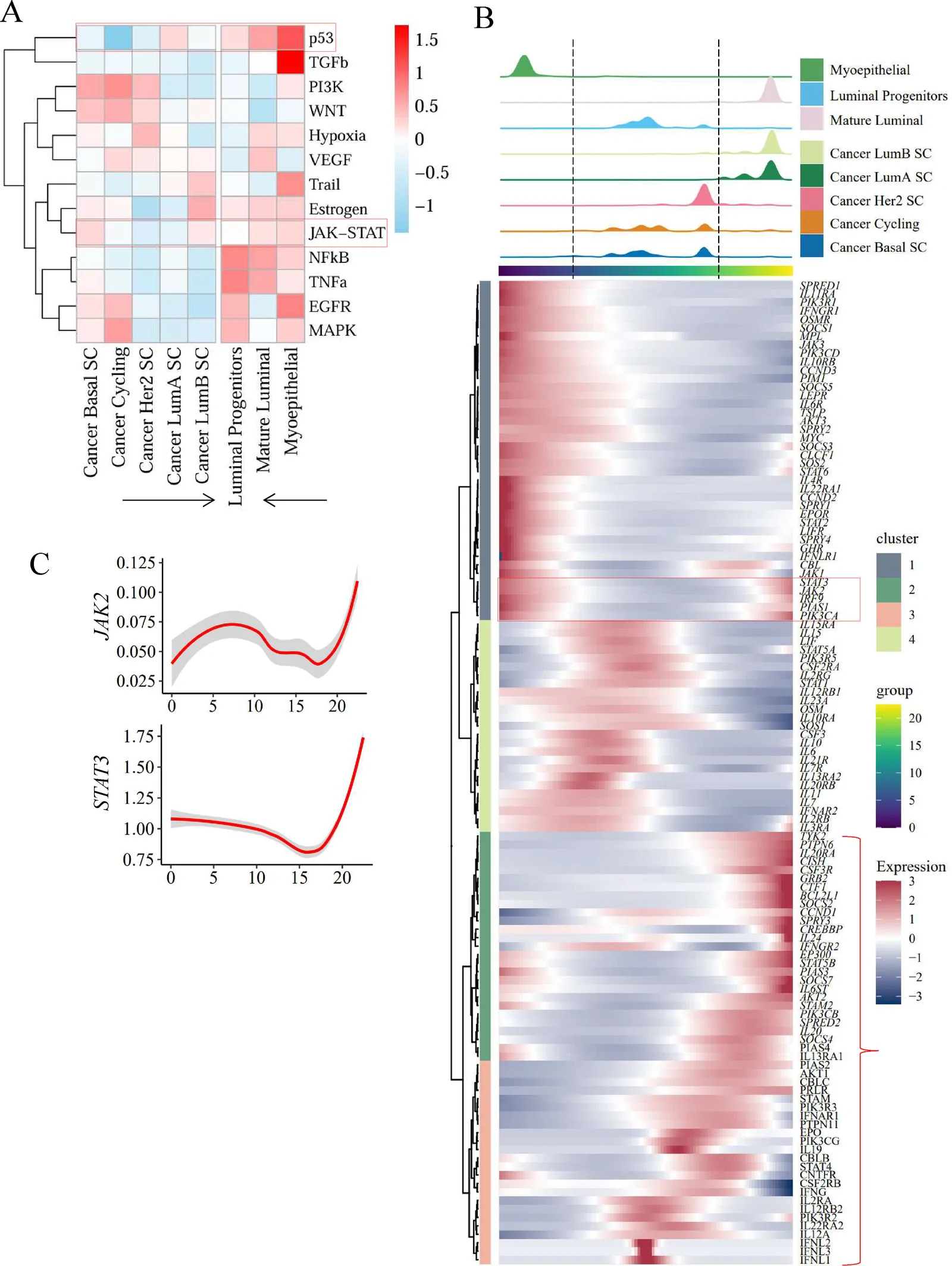
Progeny pathway activity and Monocle2 profiling. (**A**) Progeny pathway activity of epithelial cells. (**B**) Pseudotime trajectory analysis in epithelial cells: heatmap of JAK-STAT pathway (JSP) gene expression. Red brackets: T-cell differentiation-specific genes. (**C**) Relationship between pseudotime score and gene expression (JAK2, STAT3).

## Results

### Dynamic shift of JSP in carcinomas

In the TCGA pan-cancer cohort, JSP is typically higher in adjacent non-tumor tissues than in tumor tissues, and patients with elevated JSP expression generally experience poorer prognoses. Interestingly, in breast cancer, patients with high JSP scores display better survival outcomes, which challenges the conventional view of JSP as an oncogenic pathway (Figure 1—figure supplement 1). This indicates a heterogeneous role for the JSP in breast cancer. We next analyzed the primary breast cancer single-cell dataset (GSE176078, 26 cases) to examine the expression patterns and dynamic features of the JSP at single-cell resolution. JSP scores were calculated using the single-sample gene set enrichment analysis (ssGSEA) method, which revealed predominant expression in T cells (Figure 1B–E). Further annotation indicated elevated JSP scores within NK cells, CD4^+^ T cells, and CD8^+^ T cells (Figure 1F). Using the Ro/e algorithm, we assessed the preferential distribution of JSP across various subpopulations, finding that cells with high JSP scores were primarily enriched in T cells, while paracancerous epithelial and tumor cells showed significantly lower enrichment (Figure 1G and H). Pseudotime analysis illuminated the developmental trajectory from paracancerous epithelial cells to tumor cells (Figure 1I–L, Figure 1—figure supplement 1). During this progression, JSP demonstrated a dynamic shift, initially decreasing and then increasing, suggesting differential biological functions and dynamic dependencies between paracancerous and tumor epithelial cells (Figure 1L), which is consistent with previous reports: basal cells differentiated into luminal cells in breast cancer (STAT3 serves as a crucial transcription factor in basal cells) (Nguyen et al., 2018); tumorigenesis from basal cells to luminal cells depended on STAT3 in prostate cancers (Feng et al., 2025). Progeny pathway activity analysis corroborated this trend, underscoring greater JSP activity in epithelial cells and heterogeneity during tumor cell differentiation (Figure 1M). These findings demonstrate that the JSP exhibits heterogeneous expression patterns and dynamic transitions within the breast tumor cells and epithelial cells.

### JSP facilitates tumor malignant phenotypes and immunosuppression

**Figure 2.**
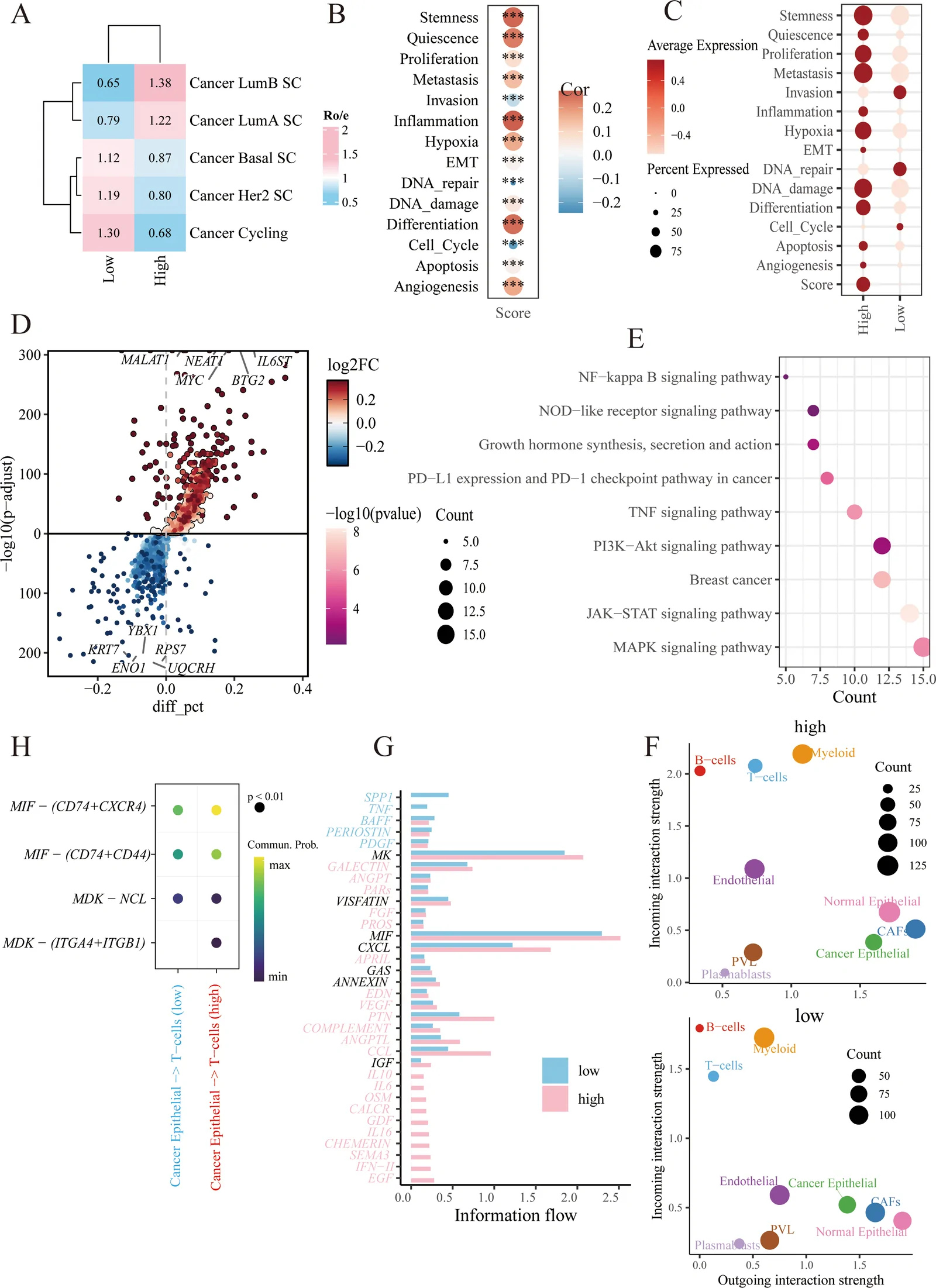
The JAK-STAT pathway (JSP) promotes tumor malignant phenotypes and immunosuppression. (**A**) Ro/e index analysis demonstrates the preferences of JSP-high versus JSP-low cells in tumor cells. (**B**) Correlation of JSP scores with 14 cancer-related phenotype features. (**C**) Expression of cancer phenotype signatures in JSP-high and JSP-low tumor cells. (**D**) Differentially expressed genes in JSP-high versus JSP-low tumor cells. (**E**) KEGG enrichment analysis of differentially expressed genes. (**F**) CellChat analysis illustrating the strength of cellular communication in JSP-high and JSP-low samples. (**G**) Bar chart of CellChat signaling flows. (**H**) Cell-to-cell communication between cancerous epithelial cells and T cells.

Next, we analyzed the potential biological functions of the JSP in tumor cells and its interplay with the immune microenvironment. Within tumor cell subgroups, JSP was enriched in the LumA and LumB subtypes (Figure 2A). Correlation analysis revealed a positive association between JSP and various cancer characteristic scores, including proliferation, invasion, and differentiation (Figure 2B). By classifying tumor cells into JSP-high and JSP-low groups based on median JSP scores, it was observed that JSP-high tumor cells exhibited a higher degree of malignancy, along with elevated expression of these characteristic scores (Figure 2C). Subsequently, we performed differential gene analysis to uncover potential biological functions of JSP in tumor cells. Oncogenes, such as *MYC*, *IL6ST*, and *NEAT1*, were found to be upregulated in JSP-high tumor cells (Figure 2D). Pathway enrichment analysis indicated that these differential genes are enriched in oncogenic signaling pathways such as *MAPK*, *NF-κB*, and *CD274/PDCD1* immune checkpoints (Figure 2E). Cell communication analysis revealed differences in immune microenvironment signaling intensity between JSP-high and JSP-low patients (Figure 2F). Signals such as *IL6*, *CCL*, *EGF*, and *IL10* were present only in JSP-high patients (Figure 2G). Moreover, enhanced immunosuppressive signaling such as *MIF-CD74+CXCR4* and *MIF-CD74+CD44* was observed in JSP-high patients (Figure 2H). In addition, we verified the spatial co-localization of *MIF*-expressing tumor cells and T cells using the breast cancer spatial transcriptomic dataset GSE210616 (Figure 2—figure supplement 1). These findings expand our understanding of the oncogenic role of JSP in breast cancer and its interplay within the immune microenvironment. JSP resonates with carcinogenic signals such as *MAPK* and *NF-κB* and is involved in immunosuppression through *MIF-CD74+CXCR4* and *MIF-CD74+CD44* signaling pathways.

**Figure 2—figure supplement 1.**
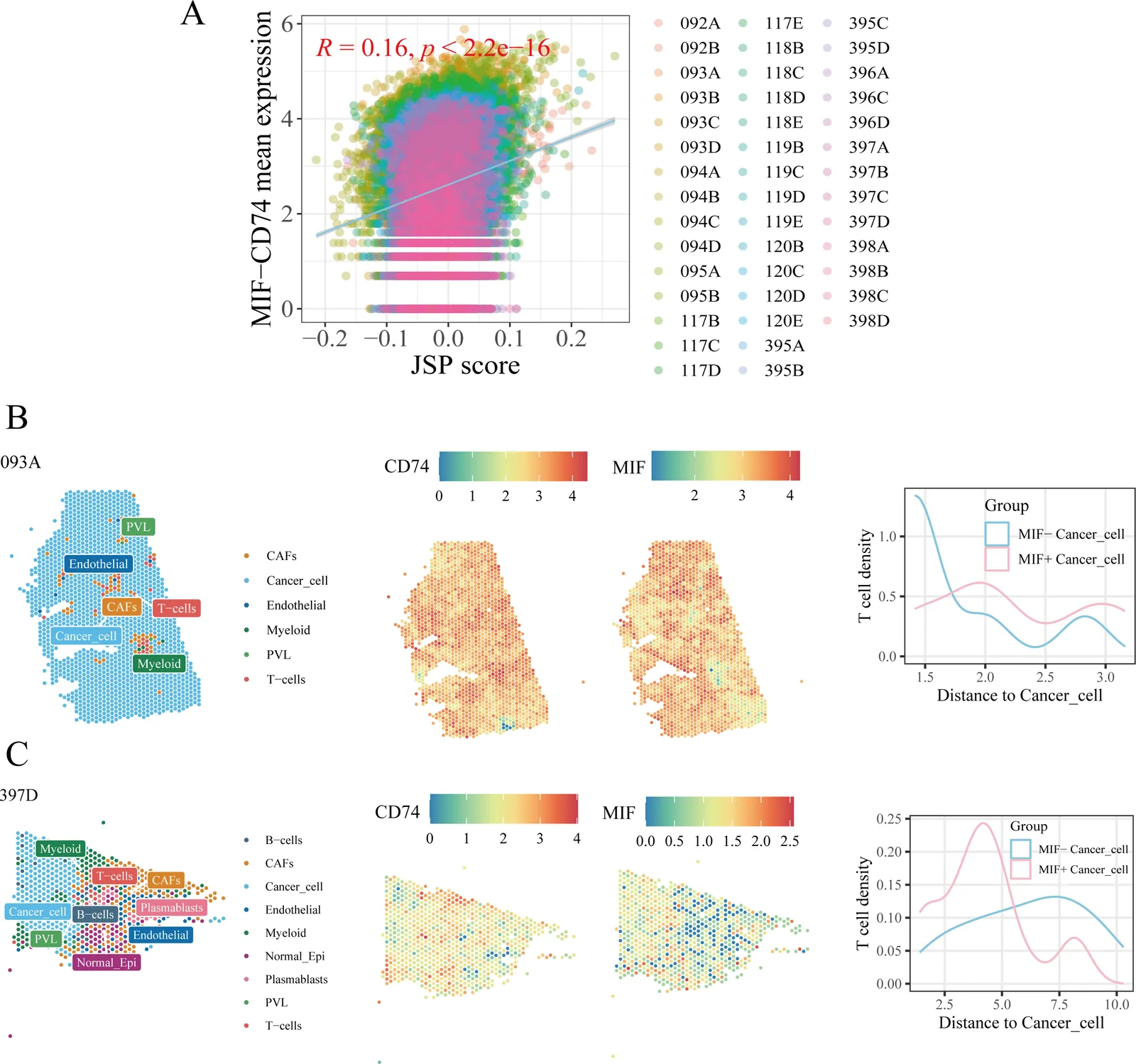
Spatial transcriptomic analysis validates the MIF-CD74 axis. (**A**) Correlation between the mean expression of MIF-CD74 and JAK-STAT pathway (JSP) score. (**B, C**) Representative cases 093A (**B**) and 397D (**C**) from the GSE210616 dataset. Left panels: cell-type annotation; middle panels: spatial expression of MIF and CD74; right panels: spatial neighborhood analysis of MIF^+^ and MIF⁻ tumor cells.

### JSP is essential for breast epithelial cell differentiation

**Figure 3.**
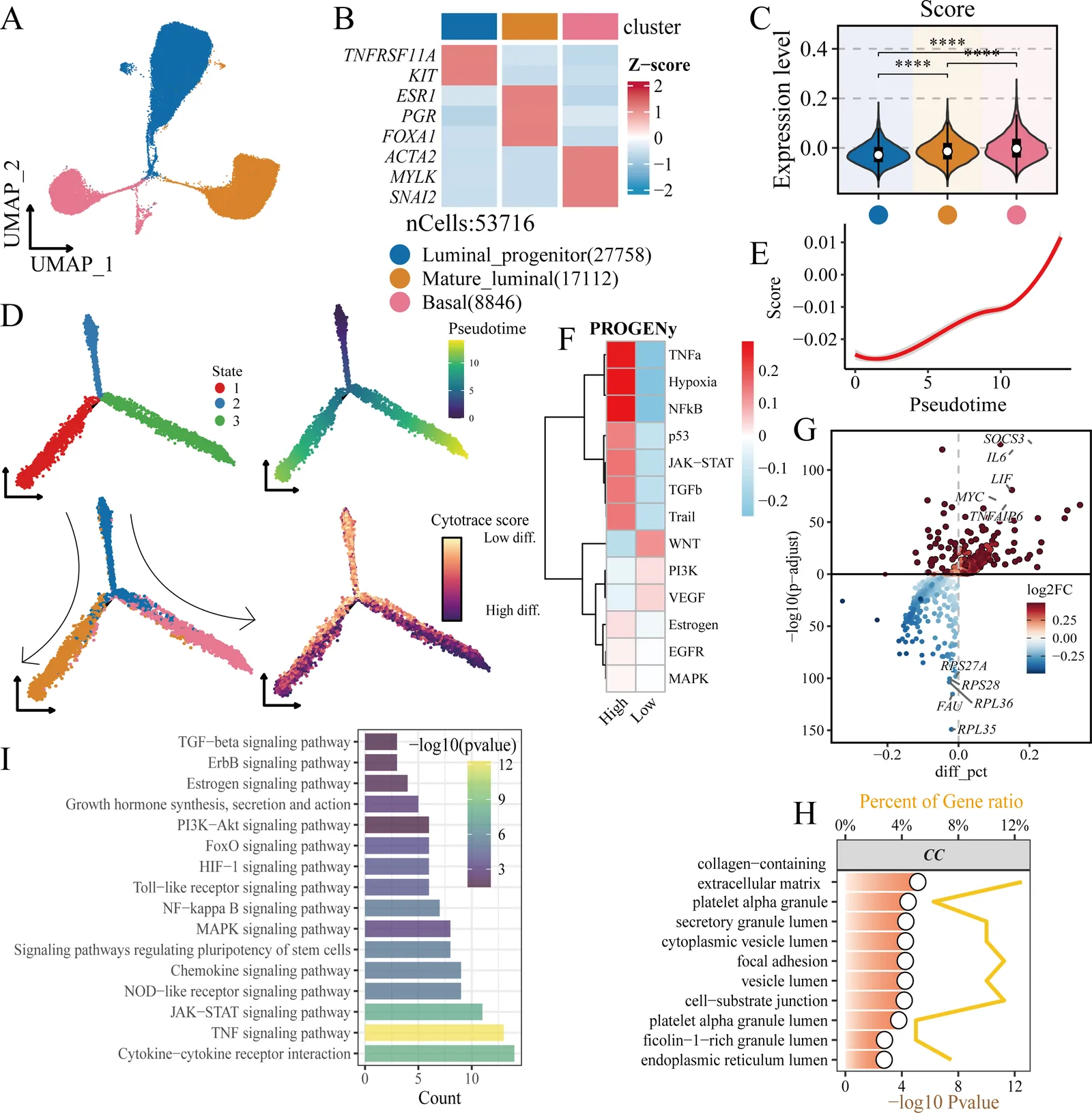
JAK-STAT pathway (JSP) is essential for normal breast epithelial cell differentiation. (**A**) UMAP plot of annotated normal epithelial cells. (**B**) Core markers of mammary epithelial cells. (**C**) Expression levels of JSP score across mammary epithelial cell subgroups. (**D**) Pseudotime trajectory analysis using Monocle2. (**E**) Relationship between JSP expression and pseudotime scores. (**F**) Progeny pathway activity, with epithelial cells categorized into high and low groups based on median JSP expression. (**G**) Volcano plot of differentially expressed genes. (**H, I**) GO and KEGG enrichment analyses of differentially expressed genes. Significance: *p<0.05; **p<0.01; ***p<0.001.

Given the differential expression patterns of JSP in adjacent non-cancerous and tumor epithelial cells (Figure 1D–F), we analyzed another human normal breast epithelial single-cell dataset (GSE161529) to explore the role of JSP in breast epithelial cell differentiation under normal physiological conditions. JSP showed higher expression in basal epithelial cells (Figure 3A–C). Pseudotime analysis revealed the differentiation trajectory of epithelial cells: from luminal progenitor to mature luminal and basal cells (Figure 3D, Figure 3—figure supplement 1A). During epithelial cell differentiation, JSP expression increased (Figure 3E). In JSP-high epithelial cells, enhanced activity of the p53 and TGFβ pathways was observed (Figure 3F). Differential gene analysis and GO enrichment for JSP-high versus JSP-low epithelial cells indicated that JSP signaling is involved in maintaining normal breast epithelial cell functions, such as focal adhesion, vesicle lumen, and secretory granule lumen (Figure 3G and H). KEGG pathway analysis suggested that JSP is associated with several important signaling pathways in epithelial cells, including estrogen, TGF-β, and FoxO pathways (Figure 3I). These results suggest that, under physiological conditions, the normal development and functional maintenance of breast epithelial cells depend on JSP signaling.

**Figure 3—figure supplement 1.**
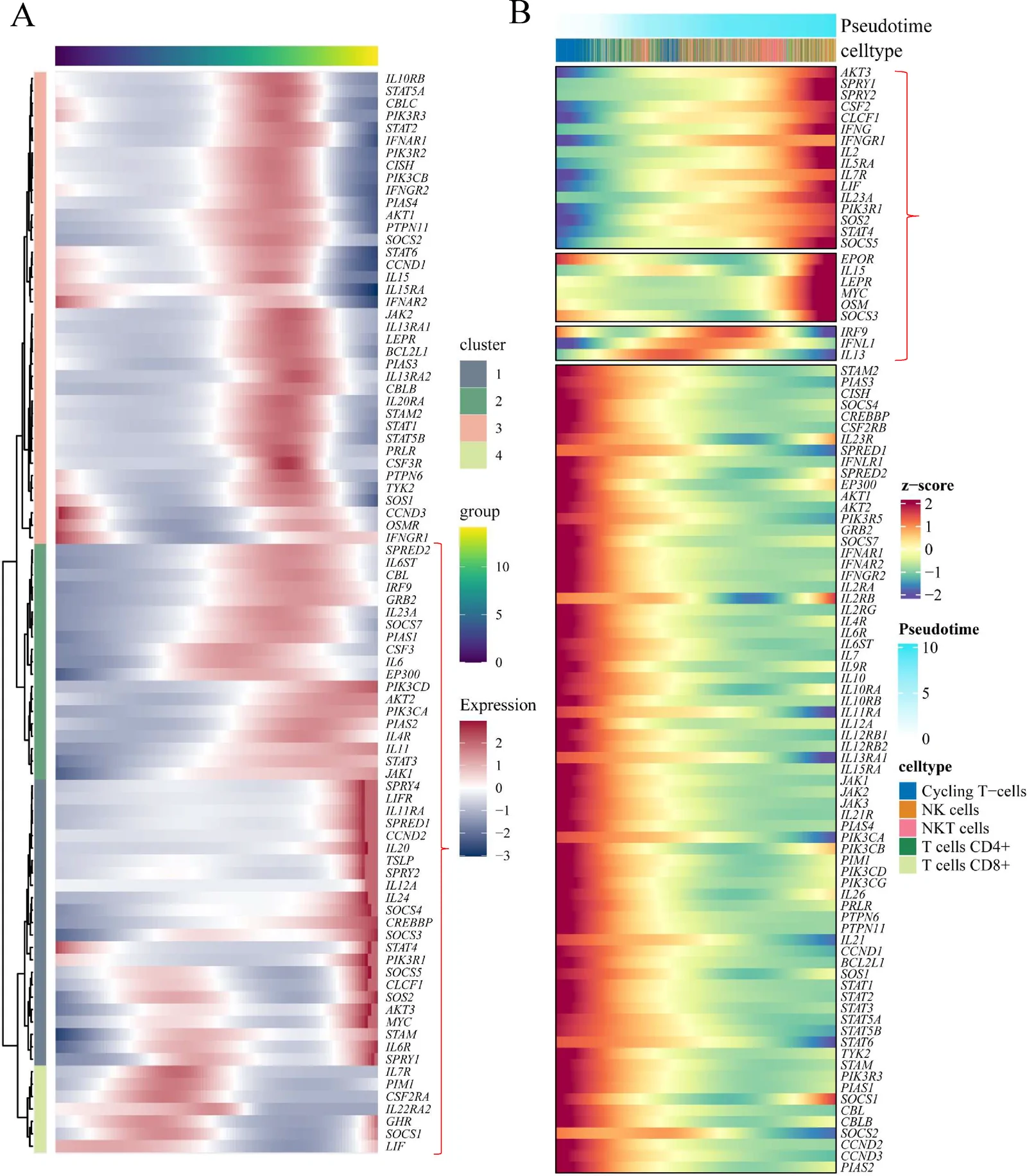
Pseudotime trajectory analysis of the JAK-STAT pathway (JSP) in normal epithelial cells and T cells. Differentiation trajectory of normal epithelial cells (**A**) and T cells (**B**). Genes associated with JSP that exhibited elevated expression during late differentiation were designated as cell differentiation-dependent genes, marked with red brackets.

### JSP mirrors anti-tumor immunity activity of T cells

**Figure 4.**
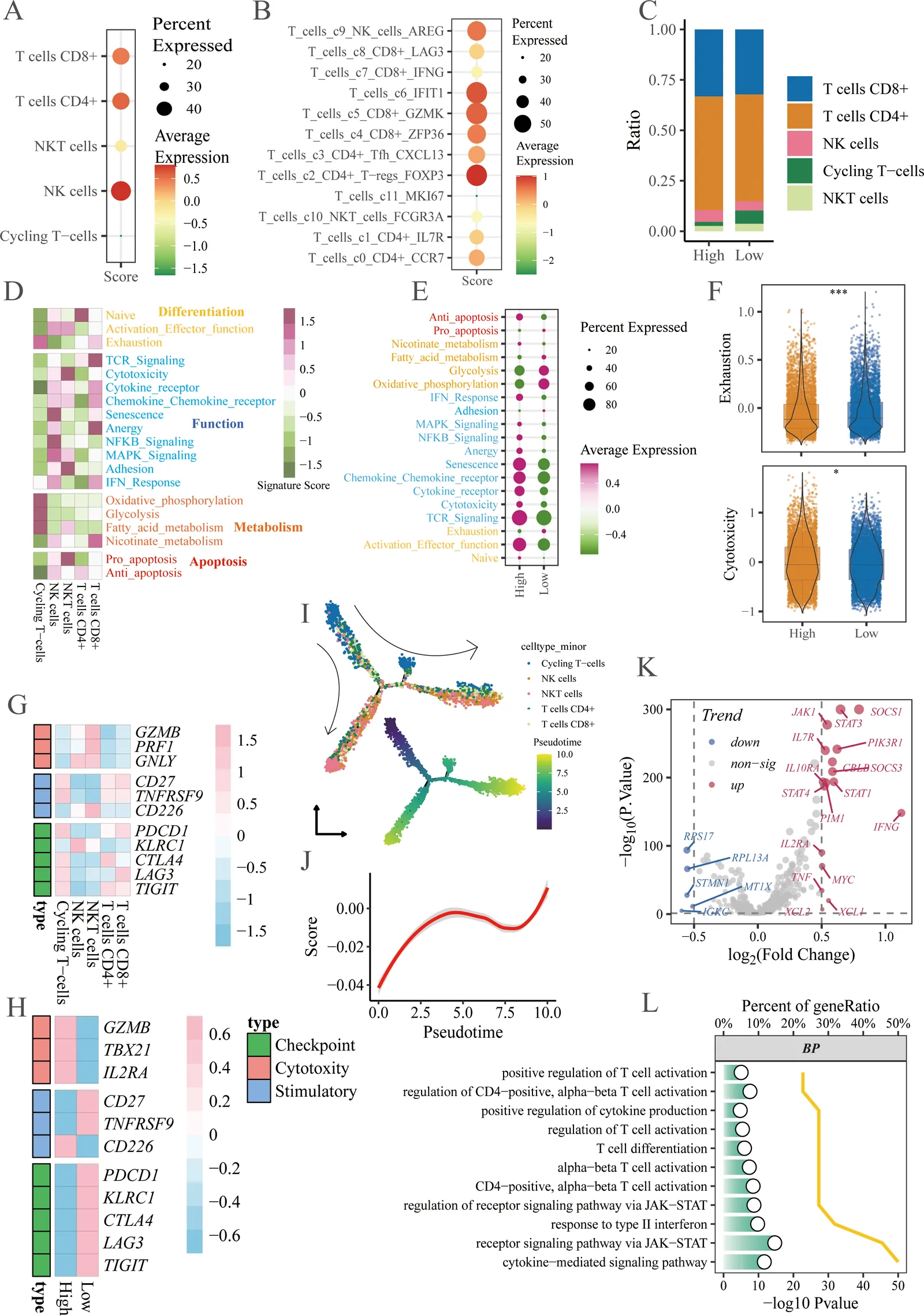
JAK-STAT pathway (JSP) is associated with enhanced T-cell function. (**A**) JSP scores across major T-cell subgroups in GSE176078. (**B**) JSP scores across minor T-cell subgroups. (**C**) Proportions of T-cell subgroups. T cells were classified into high and low groups based on the average JSP score. (**D**) T-cell state scores of major T-cell subgroups. (**E**) T-cell state scores based on JSP grouping. (**F**) Related to (**E**), exhaustion and cytotoxicity scores in T cells. (**G, H**) Heatmaps of representative functional genes in major T-cell subgroups and JSP subgroups. (**I**) Monocle2 pseudotime analysis of UMAP plots; the top panel shows cell types, the bottom panel displays pseudotime scores. (**J**) Line plot showing the relationship between JSP score and pseudotime score. (**K**) Differential genes between T-cell JSP subgroups. (**L**) GO enrichment analysis of differential genes between JSP-high and JSP-low T cells. Significance: *p<0.05; **p<0.01; ***p<0.001.

Given the highest expression of JSP in T cells (Figure 1E and F), we explored the potential biological functions of JSP in T cells. Major and minor annotations showed elevated JSP scores in CD4^+^ T, CD8^+^ T, and NK cells (Figure 4A and B). T cells were divided into JSP-high and JSP-low subgroups based on median JSP scores, with no significant differences in T-cell composition between the two groups (Figure 4C). Since the number of T cells remained unchanged, we hypothesized that JSP might impact T-cell state and function. We collected representative T-cell state signatures to comprehensively assess the status and activity of major T-cell subgroups (Figure 4D; Fu et al., 2025). JSP-high T cells exhibited increased functional scores (such as TCR signaling, IFN-γ response, cytotoxicity) and decreased metabolic scores (glycolysis, fatty acid metabolism, oxidative phosphorylation) and exhaustion scores (Figure 4E and F). Further analysis of representative functional genes in CD8^+^ T cells confirmed this, showing higher expression of T-cell cytotoxic factors (*GZMB, TBX21, IL2RA*) and lower expression of T-cell exhaustion markers (*PDCD1, KLRC1, CTLA4, LAG3, TIGIT*) in the JSP-high group (Figure 4G and H). Additionally, pseudotime analysis indicated that JSP plays an important role in promoting T-cell differentiation (Figure 4I and J, Figure 3—figure supplement 1B). Differential gene analysis revealed that JSP-high T cells exhibit higher expression of T-cell anti-tumor factors (*IFNG, TNF*: encoding TNF-α) and immune activation-related receptors (*IL2RA*: CD25, *IL10RA*) (Figure 4K). Pathway enrichment suggested that these differential genes are enriched in T-cell activation and differentiation pathways (Figure 4L). These findings collectively indicated that T cells with high expression of the JSP have stronger anti-tumor activity.

### JSP enhances immunotherapy sensitivity and predicts immunotherapy response

**Figure 5.**
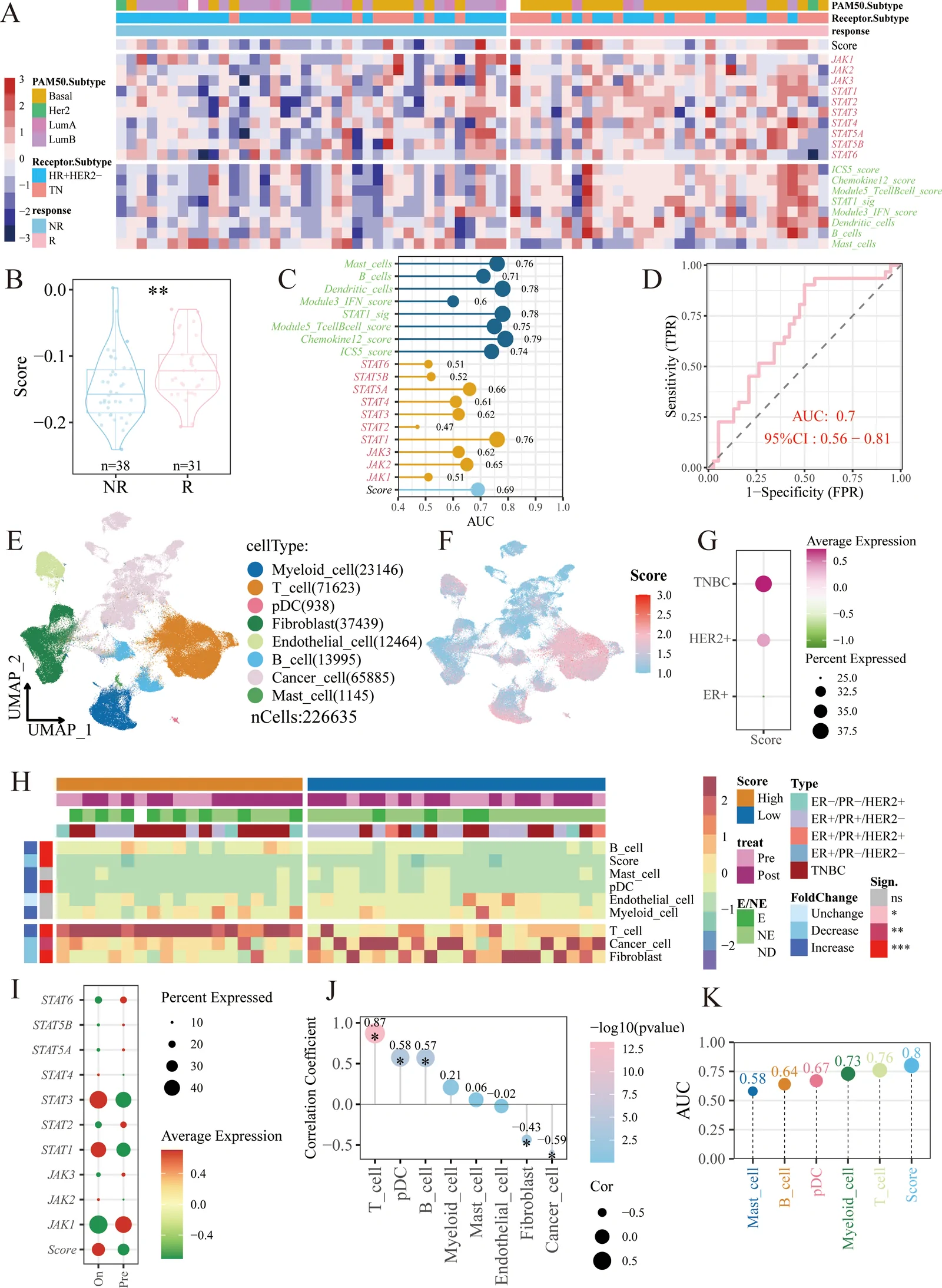
JAK-STAT pathway (JSP) enhances immunotherapy sensitivity and predicts immunotherapy response. (**A**) Expression patterns of JSP in the bulk RNA-seq immunotherapy cohort: GSE194040. Red text indicates 10 core JSP components, while green text shows 8 immune markers. TN, triple-negative breast cancer; Score, JSP score. (**B**) JSP scores in immunotherapy responders (R) versus non-responders (NR). (**C**) Area under the curve (AUC) of receiver operating characteristic (ROC) curve for predicting immunotherapy response. (**D**) ROC curve for JSP predicting immunotherapy response extracted from (**C**). (**E**) UMAP of the single-cell dataset: breast cancer anti-PD-1 immunotherapy cohort (anti-PD1_Nat.Med). (**F**) JSP score in UMAP, pDCs, plasmacytoid dendritic cells. (**G**) Distribution of JSP scores across clinical subtypes of anti-PD1_Nat.Med cohort. (**H**) Immune microenvironment characteristics (scaled cell-type ratio) of JSP-high versus JSP-low patients. (**I**) Expression of JSP and core JSP components before and during immunotherapy (Pre vs. On). (**J**) Correlation between JSP scores and cell proportions. (**K**) ROC curve AUC values: prediction of T-cell expansion using JSP scores and cell composition proportions. Significance: *p<0.05; **p<0.01; ***p<0.001.

Given that JSP is associated with T-cell cytotoxic activity, we investigated the relationship between JSP and immunotherapy response, as well as its clinical significance. In the bulk RNA-seq immunotherapy cohort (GSE194040), JSP levels were higher in responders, accompanied by an increase in immune markers and a higher proportion of TNBC samples (Figure 5A and B). JSP scores also demonstrated a relatively accurate prediction of immunotherapy response with receiver operating characteristic (ROC) = 0.7 (Figure 5C and D). To further expand the clinical applicability of the JSP score, we combined it with the mRNA expression levels of PD-L1/PD-1, which significantly improved the predictive accuracy for immunotherapy response, with the area under the curve (AUC) exceeding 0.8 (Figure 5—figure supplement 1A). Subsequently, we further explored the significance of JSP before and after immunotherapy in a single-cell immunotherapy dataset (anti-PD1_Nat.Med) (Figure 5E–K). JSP was primarily expressed in T cells (Figure 5E and F) and enriched in TNBC samples (Figure 5G). In JSP-high patients, there was a higher proportion of TNBC cases and T-cell expansion, along with improved immune infiltration, characterized by increased proportions of T cells, B cells, and plasmacytoid dendritic cells (pDCs), and a decrease in cancer cells and fibroblasts (Figure 5H). JSP showed a positive correlation with T cell, B cell, and pDC proportions, and markedly increased after immunotherapy (Figure 5I and J). Additionally, JSP was able to predict T-cell expansion more accurately compared to other immune cell proportions (Figure 5K). These results demonstrated that patients with high JSP levels exhibit a greater response rate to immunotherapy, especially in TNBC.

**Figure 5—figure supplement 1.**
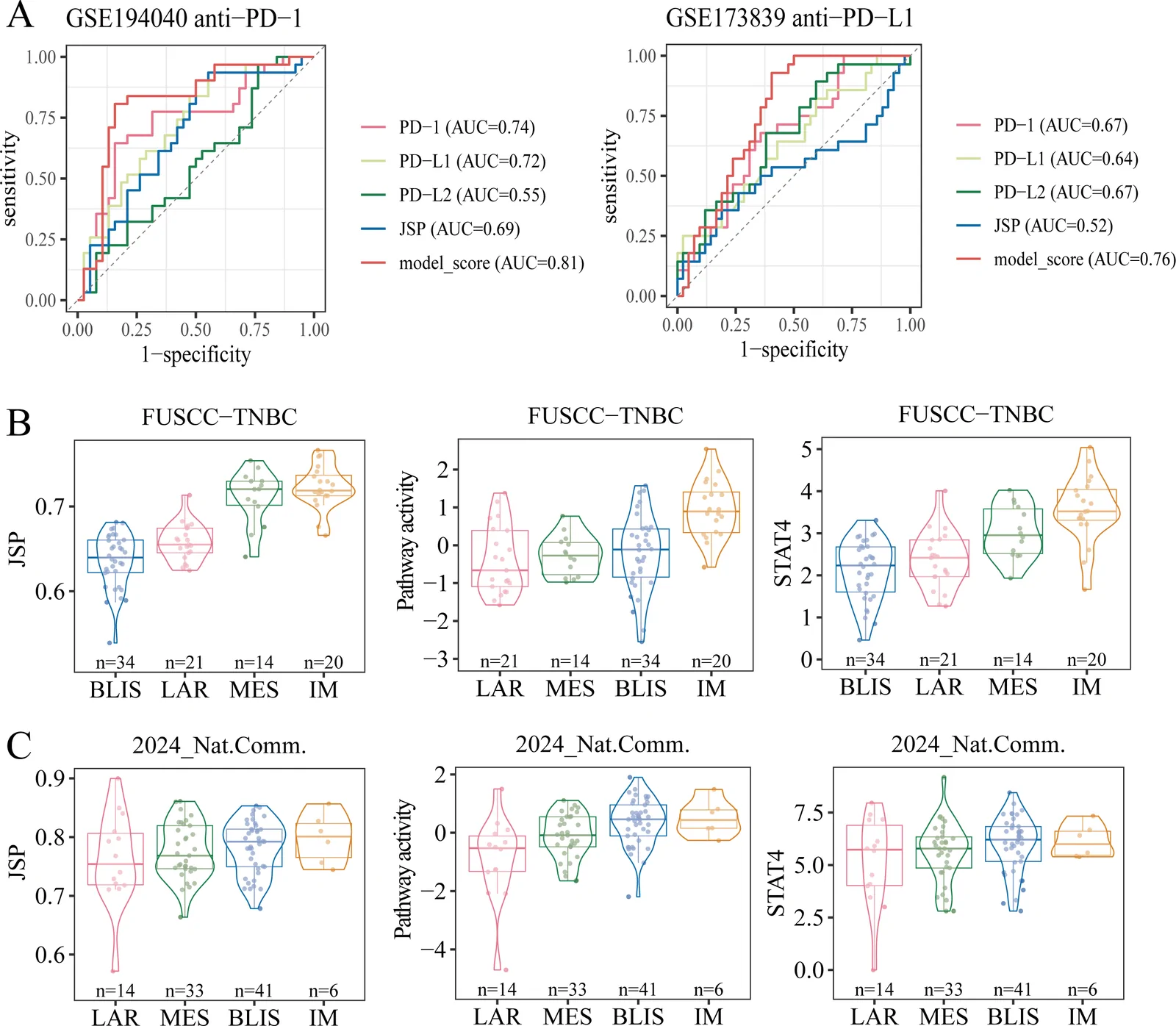
Combined predictive model and heterogeneity of the JAK-STAT pathway (JSP) in triple-negative breast cancer (TNBC) subtypes. (**A**) Receiver operating characteristic (ROC) curves of PDCD1(PD-1), CD274(PD-L1), PDCD1LG2(PD-L2), JSP score, and combined model score. (**B**) FUSCC-TNBC cohort. (**C**) 2024_Nat.Comm. cohort. Left panels: JSP scores; middle panels: JSP activity; right panels: STAT4 expression. LAR: luminal androgen receptor, IM: immunomodulatory, BLIS: basal-like immune-suppressed, MES: mesenchymal-like.

### STAT4 promotes the proliferation of cancer cells and epithelial cells

**Figure 6.**
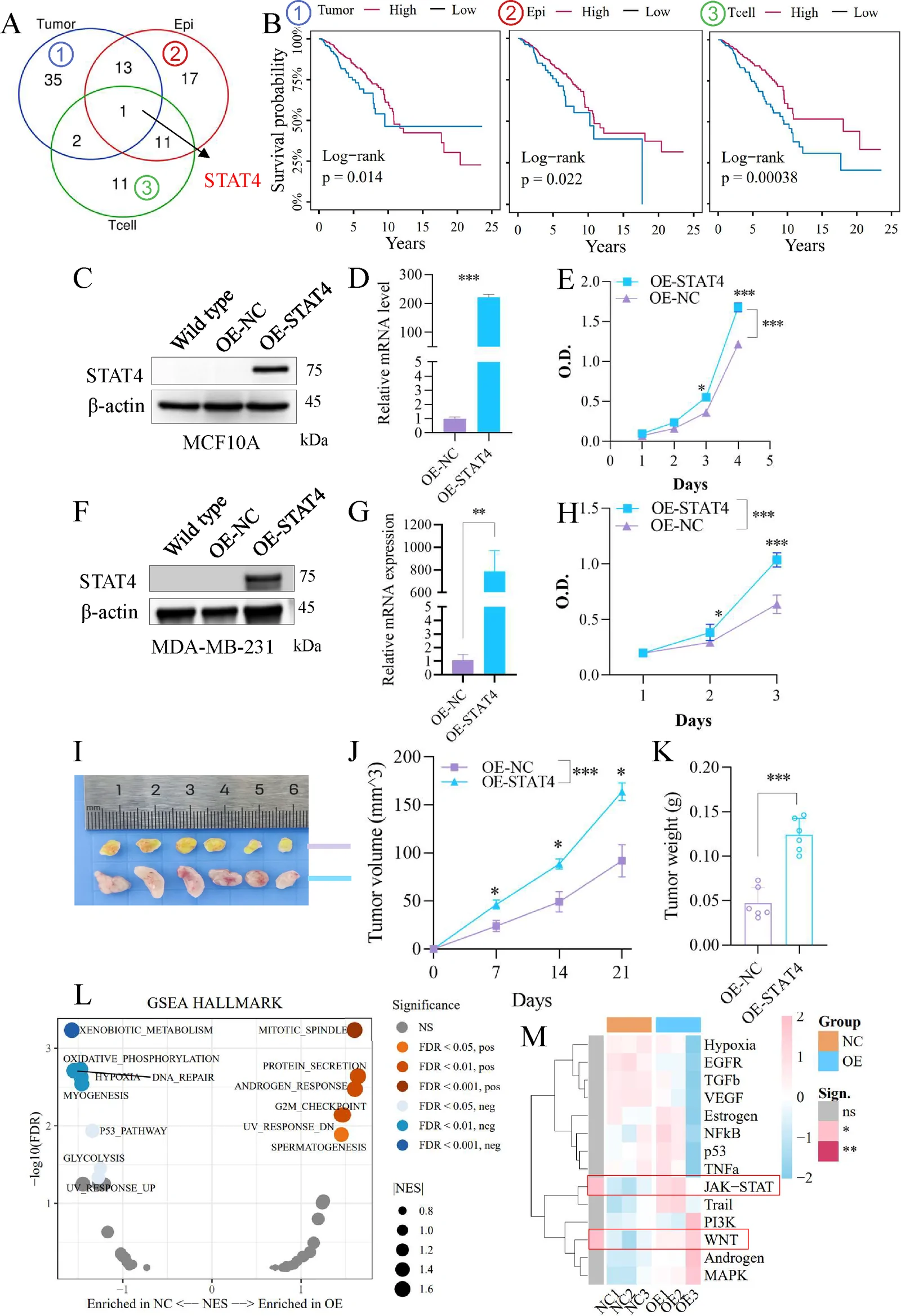
STAT4 promotes tumor cell and normal epithelial proliferation. (**A**) Venn diagram displaying the JAK-STAT pathway signature genes specific to tumor cells, epithelial cells, and T cells, which were derived from pseudotime trajectory analysis using Monocle2. (**B**) Survival analysis of the above cell-type-specific JAK-STAT pathway genes in the TCGA dataset. (**C–E**) Overexpressing *STAT4* in normal breast epithelial MCF10A cells, detected by western blotting for protein expression (**C**) and qPCR for mRNA expression (**D**), n = 3. (**E**) MCF10A cells were assigned to OE-NC (control) or OE-*STAT4* groups for cell proliferation assay, n = 3. (**F–H**) Overexpressing *STAT4* in MDA-MB-231 breast cancer cells. (**F**) Protein levels of STAT4 following *STAT4* gene overexpression. (**G**) *STAT4* mRNA levels following overexpression, n = 3. (**H**) Cell proliferation assessed by CCK-8 assay, n = 3. (**I–K**) Xenograft tumor experiments in nude mice: tumor volume (**J**) and weight (**K**), n = 6. (**L**) Gene set enrichment analysis (GSEA) of RNA-sequencing after STAT4 overexpression. (**M**) Progeny pathway activity analysis. Significance: *p<0.05; **p<0.01; ***<0.001. Figure 6—source data 1.PDF file containing original western blots for Figure 6, indicating the relevant bands and treatments. Figure 6—source data 2.Original files for western blot analysis displayed in Figure 6.

Given the heterogeneous expression pattern in tumor cells, epithelial cells, and T cells, we obtained these cell-type-specific genes from pseudotime trajectory analysis (Figure 6A, Figure 1—figure supplement 2B, Figure 3—figure supplement 1). Tumoral JSP score was negatively associated with patient survival, while epithelial cell and T-cell JSP scores were related to favorable prognosis (Figure 6A, Supplementary file 1B). Given STAT4 protein, one of the core JSP numbers, performed a similar expression pattern with JSP (high expression is associated with favorable prognosis and is primarily observed in T cells) (Figure 6—figure supplement 1), we selected *STAT4* as an interest gene to explore its role in tumor cells and epithelial cells. In MDA-MB-231 breast cancer cells, overexpression of *STAT4* promoted proliferation, tumor growth, migration, and invasion (Figure 6C–E, Figure 6—figure supplement 2A and B). Similar results were also observed in normal epithelial MCF10A cells (Figure 6F–H). RNA-seq of MDA-MB-231 cells indicated that *STAT4* enhances G2M checkpoint and mitotic pathways (Figure 6L). *STAT4* co-activates the JAK-STAT and WNT pathways (Figure 6M). KEGG enrichment analysis suggests that STAT4 is involved in the ferroptosis pathway. Given that STAT4 protein is a potential upstream transcription factor of the ferroptosis inhibitor SLC47A1 (Lin et al., 2022; Zhou et al., 2025), we confirmed that STAT4 upregulates SLC47A1 protein expression in breast cancer both in vivo and in vitro (Figure 6—figure supplement 2). STAT4 protein directly binds to the *SLC47A1* DNA promoter to promote its transcription (Figure 6—figure supplement 3). Altogether, these results implied that STAT4, as a key transcriptional orchestrator of JSP, facilitated malignant and epithelial cell proliferation and function.

**Figure 6—figure supplement 1.**
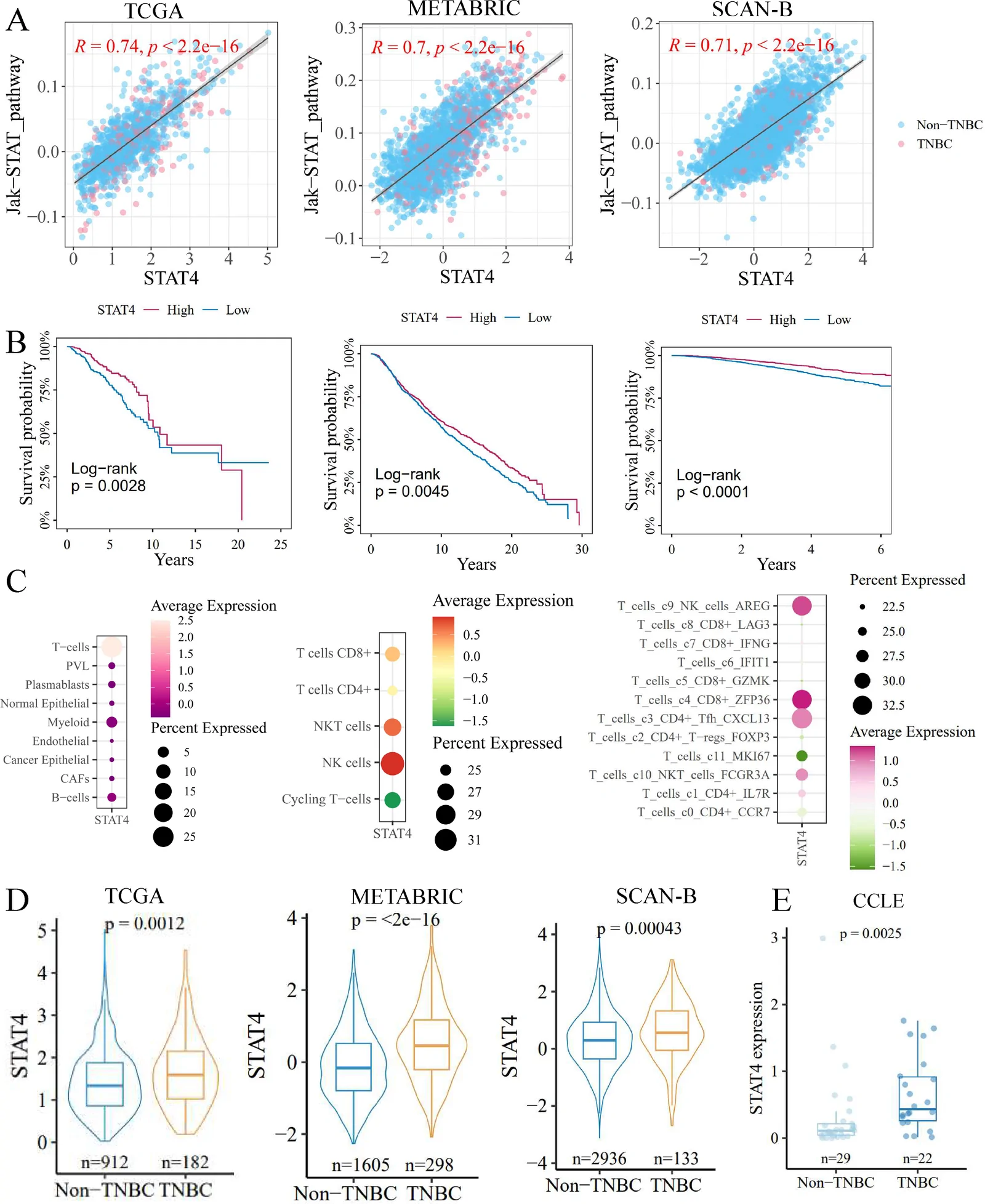
STAT4 as a representative of the JAK-STAT pathway. (**A**) Correlation between STAT4 expression and the JAK-STAT pathway score across the TCGA, METABRIC, and SCAN-B cohorts. (**B**) Survival analysis of STAT4 within these cohorts, using the median as the threshold. (**C**) Expression patterns of STAT4 in a breast cancer single-cell dataset (GSE176078), highlighting major subtypes, T-cell minor subtypes, and T-cell subsets. (**D**) Variation in STAT4 expression between triple-negative breast cancer (TNBC) and non-TNBC samples. (**E**) Expression of STAT4 in breast cancer cell lines, categorized into TNBC and non-TNBC subgroups.

**Figure 6—figure supplement 2.**
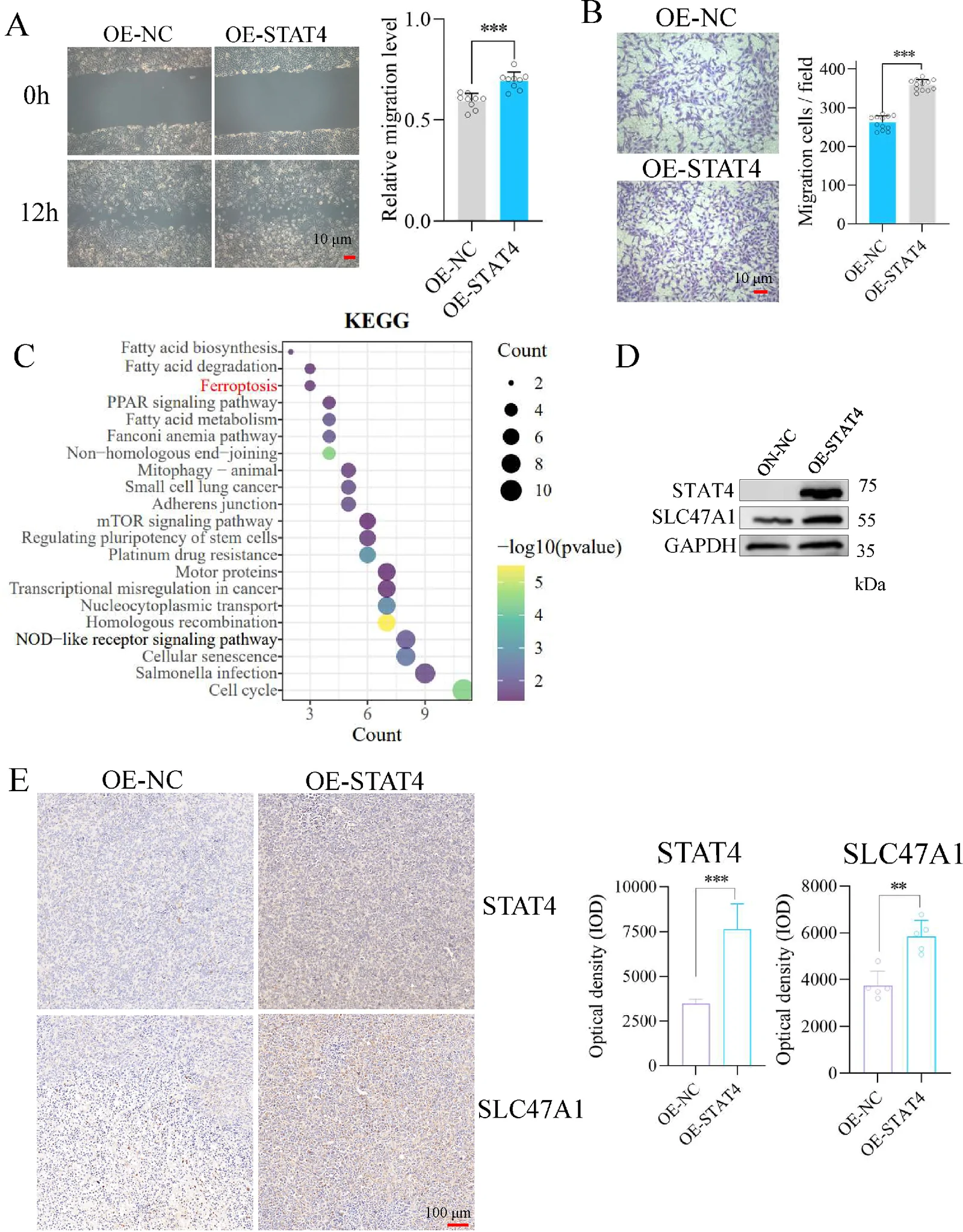
STAT4 upregulates SLC47A1 expression to involve ferroptosis in breast cancer cells. (**A, B**) Wound healing (**A**) and cell migration assays (**B**) were performed following *STAT4* overexpression in MDA-MB-231 cells, n = 9. (**C**) KEGG pathway enrichment analysis of differentially expressed genes following *STAT4* overexpression. (**D**) Upregulation of SLC47A1 protein expression upon STAT4 overexpression. (**E**) Protein expression levels of STAT4 and SLC47A1 in xenograft tumors in nude mice, n = 6. Significance: *p<0.05; **p<0.01; ***p<0.001. Figure 6—figure supplement 2—source data 1.PDF file containing original western blots for Figure 6—figure supplement 2, indicating the relevant bands and treatments. Figure 6—figure supplement 2—source data 2.Original files for western blot analysis displayed in Figure 6—figure supplement 2.

**Figure 6—figure supplement 3.**
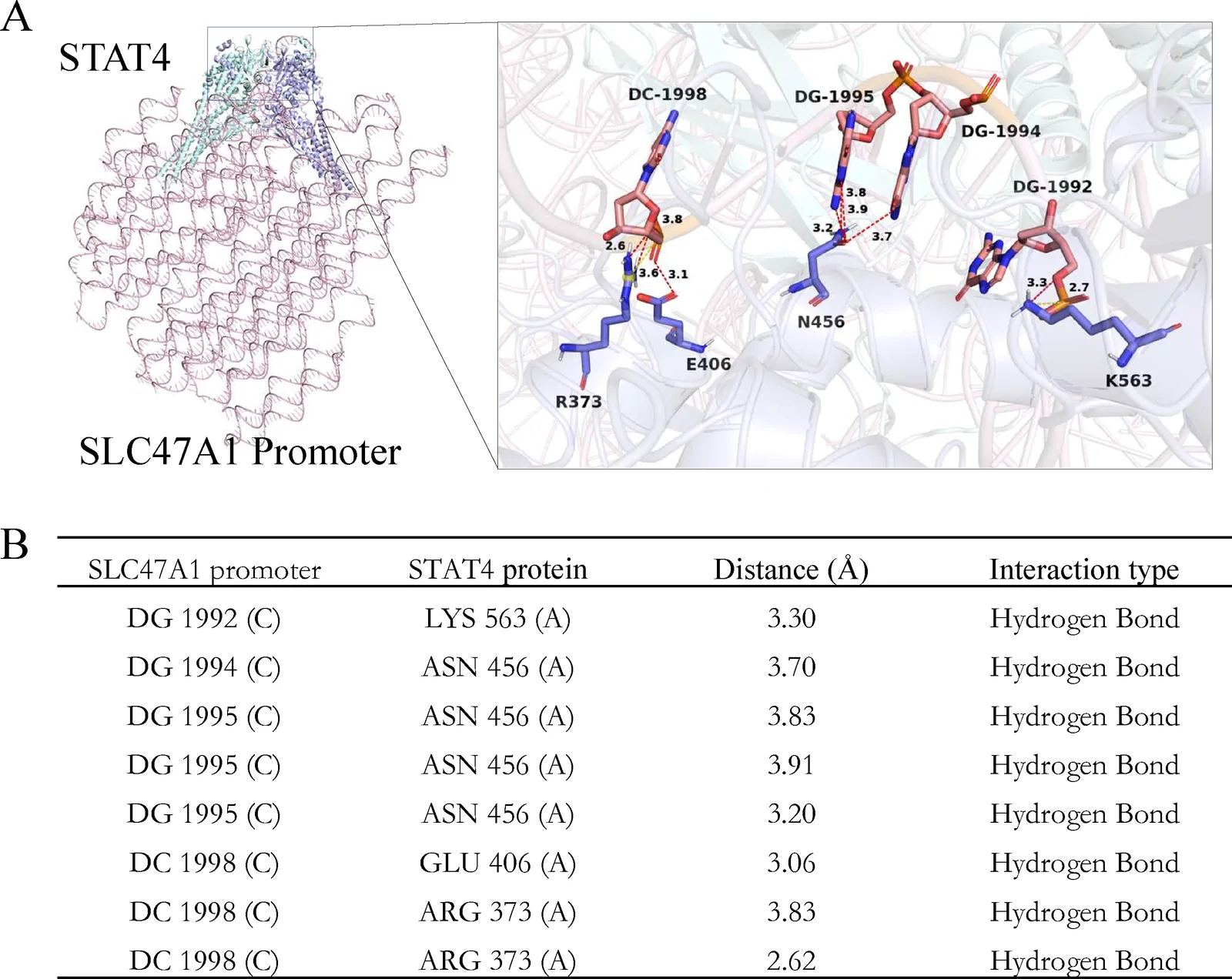
STAT4 protein binds to the SLC47A1 gene promoter. (**A**) Hydrogen bond interactions between STAT4 protein and its binding sites on the *SLC47A1* promoter, with a binding energy of −46.28 kcal/mol. (**B**) Positions and corresponding distances of these hydrogen bonds.

## Discussion

We analyzed single-cell datasets to explore the intra-tumoral heterogeneity of the JSP. Its expression was highest in T cells and lowest in epithelial cells, with normal epithelial cells expressing higher levels than tumor cells. In all epithelial cells containing normal epithelial and tumor cells, JAK-STAT signaling was reprogrammed during basal-to-luminal differentiation and tumor formation, showing an initial decrease followed by an increase. Its activity declines during the transition from normal epithelium to early tumors, then rises in advanced tumor stages due to tumor subtype heterogeneity and subtype-specific dependency. In tumor cells, higher JAK-STAT activity correlated with enhanced pro-tumor functions (e.g. migration, invasion, proliferation) and immunosuppressive signaling through the MIF-CD74 axis. Recent studies identify a conserved MIF-CD74/CD74^+^ lipid-associated macrophages axis driving T-cell exhaustion and multi-organ metastasis in breast cancer, correlating with poor survival (Insua-Rodríguez et al., 2026). Our findings reveal that tumor-intrinsic JAK-STAT signaling may depend on a similar immunosuppressive cascade, while STAT4 activation in T cells reverses immune suppression. Combining MIF-CD74 blockade with targeted STAT4 activation may synergistically remodel the metastatic niche and improve immunotherapy efficacy in TNBC. To exclude tumor background, we used single-cell data from normal human mammary epithelial cells to assess the role of JAK-STAT signaling in differentiation, finding that epithelial differentiation depends on JSP, potentially working with TNF and TGF-β signaling to maintain luminal integrity and secretory function. Meanwhile, JSP is involved in T-cell differentiation, representing enhanced cytotoxicity and functionality of terminal and effector-like T cells. To apply our findings, in a bulk RNA-seq immunotherapy cohort, higher JSP levels correlated with better treatment response, increased immune markers, with accessible accuracy for response prediction. Single-cell analysis confirmed JSP enrichment in T cells, with enhanced immune infiltration and T-cell expansion after post-immunotherapy. In brief, high JSP levels predict better immunotherapy response, particularly in TNBC. Our findings suggest that baseline JSP activity could serve as a potential biomarker for predicting the efficacy of PD-1 immunotherapy in breast cancer. This discovery holds promise for advancing precision medicine by improving response rates and reducing treatment-associated toxicity (Rayson et al., 2024). In survival profiling, higher T cell- and normal epithelial-specific JSP scores correlate with favorable patient survival, whereas elevated tumor-intrinsic JSP scores are associated with poor prognosis. This can be attributed to the predominant expression of JSP in T cells, which enhances T-cell-mediated anti-tumor immunity and counterbalances its pro-tumor effects within cancer cells.

**Figure 7.**
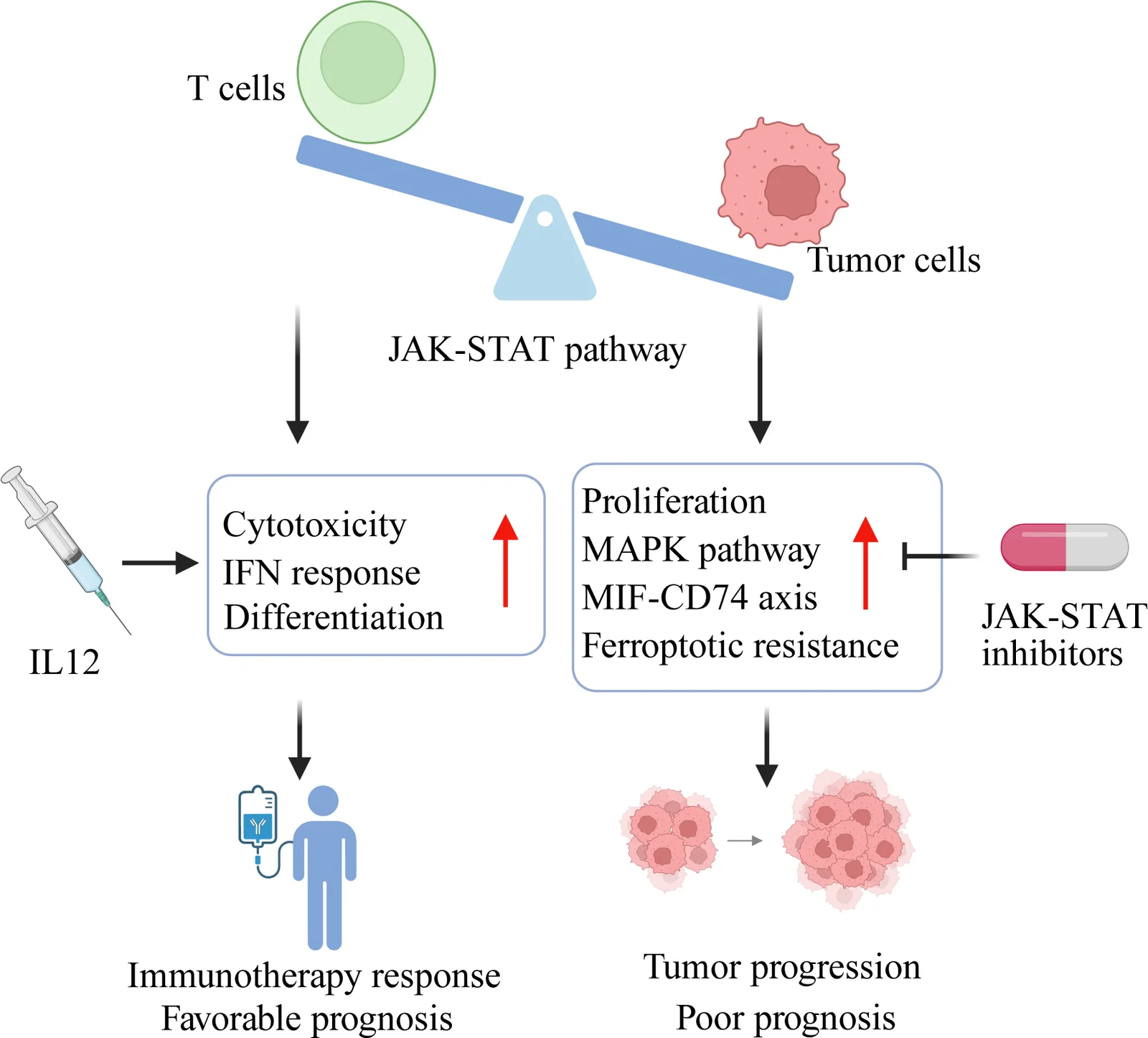
Graphical summary. Elevated JAK-STAT activity drives malignant progression and adverse outcomes in tumor cells, while JAK-STAT signaling within T cells facilitates anti-PD-1 immunotherapy responses and superior survival.

STAT4 exhibits contrasting roles in tumor cells and immune cells, highlighting its dual nature in cancer biology. On one hand, STAT4 in tumor cells drives carcinoma progression. For example, STAT4 has been shown to transactivate KDM5D, which erases H3K4me2/3 and H3K27ac marks, leading to the repression of cell-cell junction genes and antigen presentation genes. This, in turn, facilitates cancer metastasis and immune evasion in colorectal cancer (Li et al., 2023a). Additionally, STAT4 contributes to the progression of TNBC by co-transactivating with STAT3 to establish the IL12R/JAK2-STAT3-STAT4/PD-L1 axis (Zhou et al., 2023). We also confirmed that STAT4 facilitated cancer progression through orchestrating with JSP and involved ferroptotic resistance through upregulating SLC47A1. On the other hand, STAT4 plays a critical role in maintaining the anti-tumor efficacy of immune cells within TME. Recognized as a favorable prognostic biomarker in breast cancer (Zhou et al., 2023; Núñez-Marrero, 2019; Yang et al., 2020b) and gastric cancer (Zhang and Yu, 2019), STAT4 promoted anti-tumor immunity mediated by Th1 and Th17 through increasing IFN-γ production in HNSCC (Anderson et al., 2019). It also enhanced NK and NKT cell cytotoxicity, promoted Th1 cell proliferation and differentiation in an IL12-dependent manner (Yang et al., 2020a; Cui et al., 2025; Landoni et al., 2024), and strengthened IFN-γ-mediated macrophage-T-cell crosstalk (Anderson et al., 2019; Xiao et al., 2023). Here, our bioinformatics analysis and experimental findings further reinforce the aforementioned perspective: STAT4 accelerates tumor cell progression and normal epithelial proliferation, while in T cells, it serves as an indicator of enhanced T-cell cytotoxicity (Figure 7).

Preclinical studies identified the potential of targeting JSP for cancer treatment through small molecular inhibitors (Wang et al., 2024b; Zhou et al., 2024; Zhou et al., 2022; Yao et al., 2025). Furthermore, a series of clinical trials unveil that JAK or STAT inhibitors perform synergistic potential toward anti-PD-1 immunotherapy in cancers (Wang et al., 2024b; Chen and Wang, 2024). For instance, STAT inhibitors (BBI608, Fludarabine, Celecoxib) or JAK inhibitors (ruxolitinib, itacitinib) combined with PD-1/PD-L1 inhibitors (pembrolizumab, nivolumab, toripalimab) for phase I/II clinical trials (Chen and Wang, 2024). Meanwhile, STAT3 hinders CD8^+^ T-cell recruitment to TME through suppressing IFN-γ-CXCL10 (chemoattractant for T cells)-CXCR3 (CXCL10 receptor) signaling in lung cancer preclinical models, which implied targeting STAT3 in T cells could also enhance T-cell infiltration, even immunotherapy efficiency (Yue et al., 2015; Reschke and Gajewski, 2022). Moreover, STAT3 is one of the upstream transcriptional factors of CD274 (coding PD-L1) to involve PD-L1-mediated immune evasion (Jin et al., 2024; Cha et al., 2019). Targeting JAK/STAT-PD-L1 crosstalk is regarded as a druggable strategy to boost immunotherapy in lung cancers (Yao et al., 2025). JAK1 inhibitor (ruxolitinib) improved anti-PD-1 treatment response through reshaping T-cell differentiation in a lung cancer cohort (Mathew et al., 2024), while ruxolitinib reprogrammed exhausted T cells in Hodgkin’s lymphoma (Zak et al., 2024). However, the strategy of targeting the JAK-STAT signaling pathway for breast cancer treatment has proven to be both underwhelming and disappointing, with the number of related clinical trials lagging behind those for other types of cancer (Kang et al., 2025; Kohal et al., 2024; Thuya et al., 2025). JAK1/2 inhibitor ruxolitinib in metastatic TNBC cohort didn’t reach efficacy endpoint without combination with immunotherapy (Stover et al., 2018). In a clinical trial (NCT01594216) evaluating the combination of ruxolitinib and exemestane, a steroidal aromatase inhibitor, for the treatment of HR^+^ metastatic breast cancer, none of the patients achieved complete or partial response. Additionally, nearly 50% of the participants experienced anemia as an adverse effect (Makhlin et al., 2022). These findings suggest that ruxolitinib has limited utility for treating advanced breast cancer. Another clinical trial (NCT06731153) is currently underway to investigate whether the JAK1 inhibitor ivarmacitinib can overcome resistance to PD-1 inhibitor (camrelizumab) immunotherapy in TNBC. In a clinical trial (NCT03195699) evaluating the efficacy of the oral STAT3 inhibitor tinengotinib (TTI-101) as a monotherapy for advanced metastatic solid tumors, including seven cases of breast cancer, 12% of patients achieved a partial response. Among HCC patients, the overall response rate was 18%. However, adverse events or side effects of varying degrees were observed in 98% of the participants (Tsimberidou et al., 2025). Meanwhile, considering the critical role of the JSP in immune cells, its inhibition can lead to immunosuppression and immune cytotoxicity (Owen et al., 2019; Cornez et al., 2017). For instance, JAK inhibitors (such as ruxolitinib) impair the anti-tumor functions of DCs (Heine et al., 2013), NK cells (Schönberg et al., 2015), and T cells (Parampalli Yajnanarayana et al., 2015). Our analysis also partially revealed why the effectiveness of JAK/STAT inhibitors in breast cancer treatment remains unsatisfactory: broad inhibition of the JSP may hinder T-cell activation and compromise T-cell-mediated anti-tumor cytotoxicity; JSP is also crucial for normal epithelial differentiation. Considering the complexity and context-specific nature of JSP signaling and its transduction network within TME (Owen et al., 2019), leveraging the JSP as a biomarker for immune therapy response is a practical and effective approach. Based on the above clinical evidence and our findings, administering JAK-STAT inhibitors before or concurrently with immunotherapy may impair T-cell cytotoxicity and disrupt normal epithelial differentiation in breast cancer patients. Instead, sequential delivery of JAK inhibition following immunotherapy represents a promising immune-sensitizing strategy, particularly for the TNBC subtype.

Recent studies have highlighted the critical role of selecting optimal therapeutic windows for JAK inhibitors in immunotherapy. In patients with non-small cell lung cancer, intermittent treatment of JAK inhibitors during anti-PD-1 immunotherapy, rather than continuous combination therapy, has demonstrated potential for improving treatment outcomes. This approach reshapes T-cell differentiation, characterized by an increase in memory and effector-memory CD8^+^ T cells and a reduction in exhausted CD8^+^ T cells (Mathew et al., 2024). Another study in Hodgkin lymphoma found that adding JAK inhibitors at the late stage of anti-PD-1 therapy similarly increased the proportion of cytotoxic CD8^+^ T cells, accompanied by a reduction in pro-tumor neutrophils and MDSCs (Zak et al., 2024). These findings suggest that the therapeutic window should also be carefully considered when introducing JAK inhibitors into breast cancer treatment, even immunotherapy. A phase II clinical trial (NCT06731153, not yet recruiting) aims to evaluate whether the JAK1 inhibitor ivarmacitinib combined with the PD-1 inhibitor camrelizumab can overcome immunotherapy resistance in TNBC. Other clinical trial (NCT05384119) targeting palbociclib-resistant HR^+^/HER2^-^ breast cancer by combining the STAT3 inhibitor TTI-101 with established therapies to assess its safety, tolerability, and efficacy has concluded. Additionally, immune-activating cytokine IL-12 binds to its receptors IL12R-β1/β2, triggering Tyk2/Jak2 signaling to drive STAT4 phosphorylation, dimerization, and nuclear translocation; this further upregulates IFN-γ transcription to potentiate T-cell- and NK-cell-mediated anti-tumor immune responses (Ueno, 2020; Rindlisbacher et al., 2026). Several preclinical evidences have identified the efficacy of IL12 against TNBC (Hicks et al., 2021; Herpels et al., 2023). These findings suggest that combining IL-12-mediated STAT4 activation with STAT3 inhibition may open a new avenue for immunotherapy in breast cancer. Some unresolved questions include: Does JAK-STAT inhibition have therapeutic value in breast cancer immunotherapy? How can a reasonable therapeutic window be designed? Which breast cancer subtypes should be selected for further clinical trials? Is there a difference between JAK inhibition and STAT inhibition? Which is more effective? Further experiments are needed to address these questions.

In our analysis of the immunotherapy cohort, only PD-1 inhibitor datasets were included. This may limit the representativeness of our findings with respect to other ICIs, such as PD-L1 inhibitors and CTLA-4 inhibitors. Additionally, given molecular characteristics of TNBC subtype: MES (elevated JAK/STAT3 signaling pathway; Jiang et al., 2019; Asleh et al., 2022) is the abnormal activation of JAK-STAT signaling, our profiling could not refine and investigate this pathway based on these subtypes because of lack of subtype-paired single-cell cohorts. Notably, the IM subtype displayed elevated JSP expression and activity (Figure 5—figure supplement 1B and C), highlighting meaningful therapeutic implications for patients harboring this subtype. In addition, the JSP gene set may need refinement to better reflect cancer-type dependency. In summary, our results emphasize the JSP’s heterogeneity in epithelial, tumor, and T cells, with patients exhibiting high pathway activity gaining more substantial benefits from immunotherapy.

## Materials and methods

### Bioinformatical profiling

#### Data collection

The annotated human breast cancer scRNA-seq dataset (GSE176078) with 26 cases (11 ER^+^, 5 HER2^+^, and 10 TNBC) was downloaded in NCBI’s Gene Expression Omnibus: NCBI-GEO (GSE176078) and analyzed according to previous studies (Wu et al., 2021a; Zhou et al., 2023). Human normal breast epithelial scRNA-seq dataset was deposited in the NCBI-GEO database (GSE161529), and the .rds format file (SeuratObject_NormEpi.rds) was downloaded in the Figshare (https://doi.org/10.6084/m9.figshare.17058077) (Pal et al., 2021; Chen et al., 2022). The single-cell dataset of anti-PD-1 treatment (anti-PD1_Nat.Med, pembrolizumab, 29 patients: 2 ER^-^/HER2^+^, 15 ER^+^/HER2^±^, 12 TNBC) was available in the reported literature (Bassez et al., 2021). The bulk RNA-seq dataset of anti-PD-1 treatment in breast cancer was downloaded from NCBI-GEO database with the code: GSE194040 (Wolf et al., 2022).

The Cancer Genome Atlas Program-Breast Invasive Carcinoma (TCGA-BRCA, 1094 tumor tissues and 113 paired normal tissues) dataset was downloaded from GDC Data Portal (https://portal.gdc.cancer.gov/) using the TCGAbiolinks package (version 2.28.3; RRID:SCR_017683). The cleaned survival data of the TCGA cohort was obtained from the previous study (Liu et al., 2018). TCGA Pan-Cancer (PANCAN, n=11,057) was downloaded from UCSC Xena (https://xenabrowser.net/datapages/). The expression profiling and clinical data of Molecular Taxonomy of Breast Cancer International Consortium (METABRIC) dataset (2509 primary breast cancer tissues with 548 matched normal tissues) were downloaded from cBioPortal (https://www.cbioportal.org/study/summary?id=brca_metabric) (Rueda et al., 2019).

The bulk RNA-seq data of Sweden Cancerome Analysis Network-Breast (SCAN-B) dataset (3273 primary breast cancer samples) is available at NCBI-GEO database with the code: GSE96058 (https://www.ncbi.nlm.nih.gov/geo/query/acc.cgi?acc=GSE96058), while the matched clinical data was obtained from the previous literature (Staaf et al., 2019). The gene expression profiles of bulk RNA-seq with FPKM format were normalized with the log_2_(gene+1) method and duplicated samples were removed. Two publicly available TNBC RNA-seq cohorts were analyzed: the FUSCC-TNBC cohort (n=89) (Jiang et al., 2019) and the 2024_Nat.Comm. cohort (n=94) (Wang et al., 2024a).

#### Geneset scoring

The JSP with 155 genes was retrieved from GSEA database (https://www.gsea-msigdb.org/gsea/msigdb/human/geneset/KEGG_JAK_STAT_SIGNALING_PATHWAY) (Supplementary file 1A). The core JAKs-STATs pathway genes include *JAK1*, *JAK2*, *JAK3, STAT1*, *STAT2*, *STAT3*, *STAT4*, *STAT5A*, *STAT5B*, and *STAT6*. The immune-stimulating and immune checkpoint molecules, as well as cytotoxic and dysfunctional signatures, were reported in previous studies (Wu et al., 2025; Li et al., 2019). Above signatures scores were calculated using the AddModuleScore function for single-cell datasets, while ssGSEA method implemented in GSVA package (version 2.0.2; RRID:SCR_021058) was employed in bulk RNA-seq datasets.

#### Clustering and cell annotating

The normal breast epithelial cell dataset was re-annotated using the Seurat package (version 4.4.0; RRID:SCR_007322) based on following markers (Basal: *ACTA2*, *MYLK*, *SNAI2*; Luminal_progenitor: *TNFRSF11A, KIT*; Mature_luminal_cell: *ESR1*, *PGR*, *FOXA1*) (Pal et al., 2021).

#### Pseudotime and cell-cell interaction communication analysis

Monocle (version 2.28.0; RRID:SCR_016339) and CytoTRACE (version 0.3.3; RRID:SCR_022828) were applied to predict cell differentiation states and stemness in subclusters (T cells, epithelial cells from tumors, and epithelial cells of normal breast) of single-cell datasets. To explore the cellular communications, the samples were divided into Score-High or Score-Low subgroups based on the mean expression of JSP score in T cells or tumor cells. The cell-cell communication pathway and intensity were inferred using CellChat package (version 1.6.1; RRID:SCR_021946).

#### Enrichment analysis

The PROGENy pathway scores of pre-set 13 pathways were calculated using PROGENy package (version 1.22.0; RRID:SCR_006647). The differentially expressed genes were identified using the FindAllMarkers function loaded in Seurat (version 4.4.0; RRID:SCR_007322), with the default threshold: log_2_(fold change)>0.25 and p-value<0.05. The irGSEA package (version 3.3.3) was used for enrichment analysis of differentially expressed genes based on ssGSEA method. The clusterProfiler (version 4.8.3; RRID:SCR_016884) package was utilized for GO and KEGG enrichment analysis.

#### Deconvolution of immune infiltration

For bulk RNA-seq analysis, immune cell populations were inferred using the CIBERSORT algorithm, which was integrated in the IOBR package (version 0.99.9; RRID:SCR_025619) with the deconvo_tme function (Zeng et al., 2024).

#### Spatial transcriptomics

Spatial transcriptomic data were obtained from the GEO database (GSE210616), including 43 10× Visium samples from 14 primary TNBC patients (Bassiouni et al., 2023). Cell-type deconvolution was performed using the RCTD package with the scRNA-seq reference dataset GSE176078. Correlation analyses were conducted via the cor.test() function, and spatial gene expression patterns were visualized using Seurat’s SpatialFeaturePlot(). The phenoptr package (version 0.3.2; RRID:SCR_026104; https://akoyabio.github.io/phenoptr/) was used to analyze cell types spatially adjacent to MIF^+^ tumor cells.

#### Protein-nucleic docking

Protein sequences were obtained from the UniProt database, and protein-nucleic acid complex structures were predicted using ProteinX, an open-source tool with performance comparable to AlphaFold3. The predicted structures were subjected to energy optimization via the Rosetta Relax module for subsequent computational analysis. The *SLC47A1* sequence was retrieved from NCBI (https://www.ncbi.nlm.nih.gov/gene/55244/), and its promoter sequence was defined as the 2000 bp upstream genomic region. The initial 3D model of the protein-promoter complex was predicted using ProteinX, further refined, and exported in PDB format. Flexible docking was performed using RosettaDock, and candidate models were selected based on the built-in Rosetta scoring function. Protein-nucleic acid interactions and binding energies were characterized using LigPlus, FoldX, and PLIP. Conformational representations of the docked complexes were generated using PyMOL.

#### Predictive model construction

A multivariable Cox proportional hazards regression model was constructed to integrate the expression levels of *PDCD1*, *CD274*, *PDCD1LG2,* and the JSP risk score, with the aim of improving prediction of clinical response to immunotherapy in GSE194040 (I-SPY2-990, pembrolizumab, anti-PD-1) and GSE173839 (I-SPY2 trial, durvalumab, anti-PD-L1). A unified model risk score was then calculated for each sample using regression coefficients derived from the coxph() function in the R survival package (version 3.7-0; RRID:SCR_021137). To evaluate and compare predictive performance, ROC curve analysis was performed. The AUC was used as the primary index to assess the discriminative capacity of individual markers and the combined multi-marker signature. ROC curve visualization and quantitative comparison were conducted using the pROC R package (version 1.18.5; RRID:SCR_024286).

#### Survival analysis

The subcluster signatures were defined as the top 50 differentially expressed genes ranked by fold change using the FindAllMarkers function (Chu et al., 2025). Their signature score for each bulk RNA-seq was calculated using the ssGSEA method. The surv_cutpoint function of the survminer package (version 0.5.0; RRID:SCR_021094) was used to determine the optimal cutoff for these signature scores for subsequent Kaplan-Meier survival analysis with log-rank test in the survival package (version 3.7-0; RRID:SCR_021137).

### Experiments

#### Cell culture

The human TNBC cell line MDA-MB-231 was acquired from Procell System (CL-0150, Wuhan, China) and confirmed through short tandem repeat profiling alongside testing for mycoplasma contamination. Cells were cultured in complete RPMI-1640 medium supplemented with 10% fetal bovine serum (10091148, Thermo Fisher Scientific Inc, USA) and 1% penicillin-streptomycin solution (15140122, Thermo Fisher Scientific Inc, USA). MCF10A human normal breast epithelial cells (CL-0525, Procell) were cultured in DMEM/F-12 medium supplemented with 5% horse serum, 20 ng/mL EGF, 0.5 μg/mL hydrocortisone, 10 μg/mL insulin, and 1% penicillin-streptomycin. The full-length STAT4 transcript was cloned into the V5 (EF-1α/GFP & Puro) vector plasmid and verified via Sanger sequencing (Genepharma, Shanghai, China). The plasmid was co-packaged with pGag/Pol, pRev, and pVSV-G systems to produce lentiviral particles in 293T cells. After concentrating the virus and measuring its titer, MDA-MB-231 cells were infected with either the STAT4-overexpressing lentivirus or the control lentivirus for 48 hr. The cells were then subjected to puromycin selection (1 µg/mL) for 2 weeks to establish a stable cell line expressing the target gene.

#### Cell proliferation assay

CCK-8 cell proliferation assays and colony formation experiments were conducted to assess changes in tumor cell growth phenotypes following gene interference. A total of 3000 MDA-MB-231 cells from each group were seeded into 96-well plates. At 24 hr intervals, part of the medium was replaced with fresh RPMI-1640 medium containing 10% CCK-8 reagent (HY-K0301, MedChemExpress, China). The cells were incubated at 37°C for 1 hr, and optical density at 450 nm was measured using a spectrophotometer. For the colony formation assay, 1000 cells were seeded into six-well plates. Once the cells adhered, the culture medium was refreshed every 5 days. After a 14-day incubation period, the medium was removed, and the cells were fixed with 4% paraformaldehyde. The cells were subsequently stained with 0.1% crystal violet, rinsed with distilled water, and air-dried at room temperature. In the wound-healing assay, scratches were made using a 10 µL pipette tip once cell confluency in the six-well plates reached 100%. After washing the culture medium three times, images were captured. Additional images were taken 12 hr later to observe cell migration, and the migration area was quantified using ImageJ.

#### Western blotting

Total protein was extracted using RIPA lysis buffer (P0013B, Beyotime, China) and quantified using the BCA Protein Quantification Kit (ZJ102, Epizyme Biotech). A total of 30 µg of protein was subjected to SDS-PAGE for separation, after which the protein bands were transferred to a PVDF membrane. The membrane was blocked with a 5% bovine serum albumin (BSA) solution at room temperature for 1 hr, followed by incubation with primary antibodies (STAT4: #ET1701-42, dilution ratio = 1:1000, HUABIO; SLC47A1: 20898-1-AP, dilution ratio = 1:2000, Proteintech Group, Inc; GAPDH: 10494-1-AP, dilution ratio = 1:5000, Proteintech Group, Inc) overnight at 4°C. Subsequently, a secondary antibody (dilution ratio = 1:5000, SA00001-2, Proteintech) was applied at room temperature for 1 hr. The blots were visualized using an ECL Chemiluminescent Substrate Kit (36222ES60, YEASEN, China) and images were captured using a chemiluminescence imaging system (GelView 6000 Pro, BLT Photon Technology, China).

#### Real-time qPCR

Total intracellular mRNA was extracted using the TRIzol reagent (15596018CN, Thermo Fisher Scientific Inc) in accordance with the manufacturer’s instructions. The PrimeScript RT reagent Kit with gDNA Eraser (RR047A, Takara) was used to eliminate genomic DNA and reverse-transcribe RNA into cDNA. PCR amplification and fluorescence quantification were conducted using TB Green Premix Ex Taq II FAST qPCR (CN830A, Takara), with fluorescent signals collected on a real-time quantitative PCR instrument (Archimed X4, ROCGENE, China). Relative expression levels were analyzed using the 2^-ΔΔCT^ method. The primer sequences used are as follows: *STAT4* (Forward: AGCCTTGCGAAGTTTCAAGA; Reverse: ACACCGCATACACACTTGGA) and *GAPDH* (Forward: GACTCATGACCACAGTCCATGC; Reverse: AGAGGCAGGGATGATGTTCTG).

#### RNA-seq and analysis

Total RNA was extracted using TRIzol reagent, and its concentration and integrity were assessed using a NanoDrop 2000 spectrophotometer and an Agilent 2100/LabChip GX instrument. Following library construction, sequencing, and quality control of FASTQ files, HISAT2 and StringTie software were utilized to align the data to the reference genome and construct transcripts. Differential gene expression was determined from the expression matrix with fragments per kilobase of transcript per million fragments mapped (FPKM) format, employing the Limma package (version 3.56.1; RRID:SCR_010943) in R with thresholds set at p<0.05 and log_2_(fold change)>1. Gene enrichment analysis was conducted using the clusterProfiler (version 4.8.2; RRID:SCR_016884) and GSVA (version 1.48.3; RRID:SCR_021058) packages, while visualization was carried out using the ggplot2 (version 3.5.1; RRID:SCR_014601) and pheatmap (version 1.0.12; RRID:SCR_016418) packages.

#### Animal experiment

The animal experimental protocol was approved and overseen by the Medical Ethics Committee of Sichuan University (ID: K2024019). Female nude mice, procured from GemPharmatech Co., Ltd., were maintained in a specific pathogen-free animal facility at the West China School of Pharmacy, Sichuan University, with unrestricted access to food and water. A total of 2×10^6^ MDA-MB-231 cells were injected subcutaneously into the dorsal region of each mouse, with a minimum of five mice per group. Tumor volume was assessed weekly, and after 3 weeks, the mice were euthanized. Tumor tissues were excised, measured, and prepared for subsequent histological staining analysis.

#### Immunohistochemistry

Fresh tumor tissues were fixed in 10% neutral formaldehyde for 24 hr, followed by gradient dehydration, and then embedded in paraffin to create paraffin blocks. Tissue sections, 5 µm in thickness, were cut and subsequently deparaffinized and rehydrated. Peroxidase activity was inhibited using 3% hydrogen peroxide, followed by blocking with 5% BSA. The primary antibodies (STAT4: 13028-1-AP, dilution ratio = 1:200, Proteintech Group, Inc; SLC47A1: bs-9284R, dilution ratio = 1:500, Bioss Inc) were then applied and allowed to incubate overnight at 4°C. The sections were subsequently incubated at room temperature with horseradish peroxidase-conjugated anti-rabbit IgG (SV0004, Boster) and developed using a DAB kit (AR1027-1, Boster). Image analysis was conducted using Image-Pro Plus 6.0 (Media Cybernetics, Rockville, MD, USA), with integrated optical density values calculated accordingly.

### Statistics analysis

All bioinformatics analyses were performed using R (v4.3.0), and statistical analyses for experimental results based on at least three biological replicates were conducted using GraphPad Prism 9. All quantitative data are presented as the mean ± standard deviation (SD). Bioinformatic results were visualized using R software (4.4.0) with ggplot2 (3.5.1) for boxplot, pheatmap (1.0.12) and ComplexHeatmap (2.22.0) for heatmap. Linear correlation analysis was computed using the Spearman’s algorithm. The statistics significance of non-normally distributed data was calculated using the Wilcoxon signed rank test for two subgroups and Kruskal-Wallis for multiple-subgroups comparison. For normally distributed data, the unpaired t-test was used to compare the two groups, while significant differences among multiple groups were assessed using a one-way ANOVA method. A p-value of less than 0.05 was considered statistically significant.

## Data Availability

All clinical datasets are publicly available and stated in the methods section. All R codes are available on GitHub (https://github.com/Jianbo1999/2025-JAK-STAT-breast-cancer, copy archived at Zhou, 2026). Raw sequencing and immunohistochemistry (IHC) image data are deposited in Figshare, available at https://doi.org/10.6084/m9.figshare.33780247. The following dataset was generated: ZhouJ 2026Raw sequencing data and raw IHC image datafigshare10.6084/m9.figshare.33780247

## Additional files

Supplementary file 1. JSP-related gene set list. (A) JAK-STAT pathway genes. (B) Cell-type-specific JAK-STAT pathway genes.

MDAR checklist
