# Supplementary material for: JAK-STAT pathway heterogeneity governs immunotherapy response in breast cancer
Source: eLife. 2026 Oct 13;15:RP110635. doi: 10.7554/eLife.110635 (PMC13661048; doi:10.7554/eLife.110635)
Supplement: Supplementary file 1 [file elife-110635-fig6-data1.pdf]

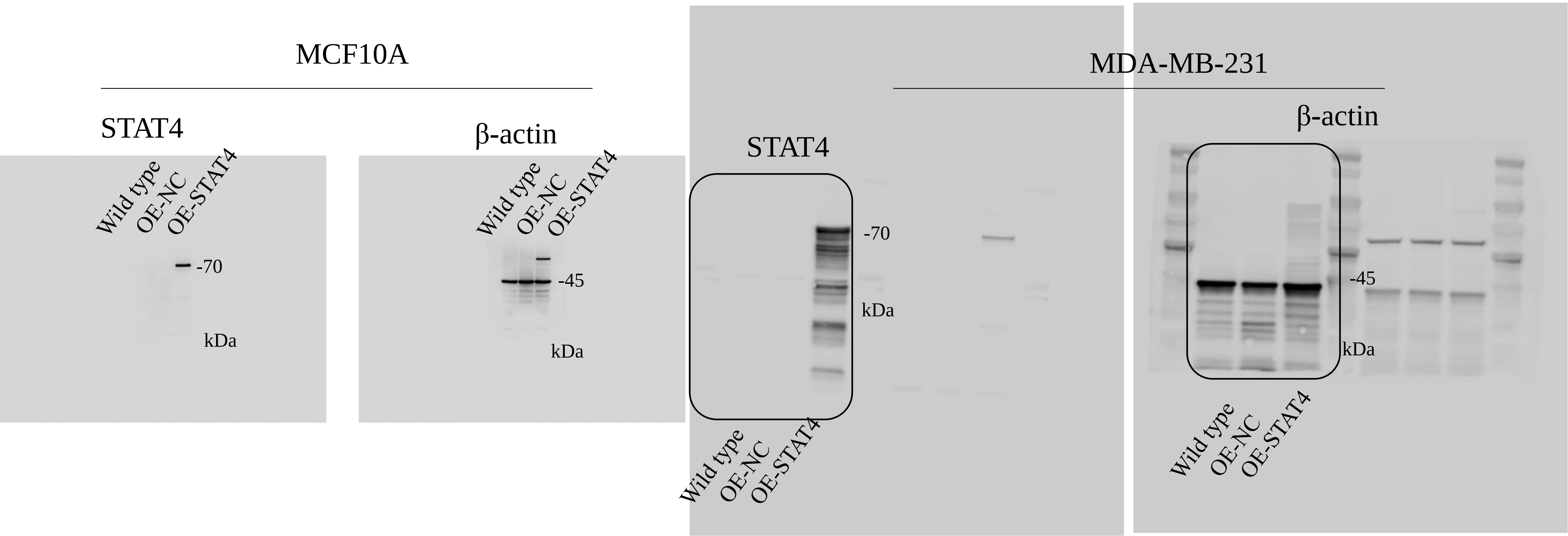

Figure 6—source data 2

Original membranes corresponding to Figure 6.
