# Supplementary material for: JAK-STAT pathway heterogeneity governs immunotherapy response in breast cancer
Source: eLife. 2026 Oct 13;15:RP110635. doi: 10.7554/eLife.110635 (PMC13661048; doi:10.7554/eLife.110635)
Supplement: Peer review history

# Editor report: eLife Assessment

**DOI:** 10.7554/eLife.110635.4.sa0

**Authors:** Yu Zhao

**Affiliations:** Institute of Radiation Medicine, Chinese Academy of Medical Sciences and Peking Union Medical College China

**Keywords:** Convincing, Fundamental

This study presents a **fundamental** finding that the JAK-STAT pathway (JSP) exerts context-dependent roles across distinct cellular compartments within the breast cancer microenvironment. The conclusions are supported by **convincing** evidence from multi-omics analyses. This study may inspire future studies to explore specific factors that selectively modulate JAK-STAT activity in immune cells to achieve favorable therapeutic outcomes.

# Referee report: Reviewer #2 (Public review)

**DOI:** 10.7554/eLife.110635.4.sa1

Summary:

The JAK-STAT pathway (JSP) exhibits cell-type-specific functional heterogeneity in breast cancer. This study investigates the JSP in breast cancer and its response to anti-PD‑1 immunotherapy. JSP displays distinct cell‑type heterogeneity: it promotes malignant phenotypes and immunosuppression in tumor cells, while enhancing cytotoxicity and reducing exhaustion in T cells. Elevated JSP expression correlates with improved immunotherapy responses, especially in triple‑negative breast cancer. These findings highlight the paradoxical roles of JSP, indicating that broad inhibition may compromise anti‑tumor immunity.

Strengths:

The major strengths of this study include the comprehensive characterization JSP heterogeneity across epithelial, tumor, and T cells in breast cancer. The identification of JSP and STAT4 as predictive biomarkers for immunotherapy response, particularly in triple‑negative breast cancer, provides clinically relevant insights for patient stratification.

Comments on revised version.

The corresponding content has been revised.

# Referee report: Reviewer #3 (Public review)

**DOI:** 10.7554/eLife.110635.4.sa2

Summary:

This multi-omics study by Zhou et al elucidates the context-dependent roles of the Janus kinase-signal transducer and activator of transcription (JAK-STAT) pathway (JSP) across different cellular compartments in the breast cancer tumor microenvironment. While bulk JSP activity is associated with a favorable prognosis, single-cell analysis reveals a paradoxical landscape: high JSP in T cells drives anti-tumor cytotoxicity and reduces exhaustion, whereas high activity in tumor epithelial cells promotes malignancy and immunosuppression via the MIF-CD74 signaling axis. The JSP score (immune-related) serves as a robust predictive biomarker for response to anti-PD-1 immunotherapy, particularly in triple-negative breast cancer (TNBC). Furthermore, the study identifies the STAT4/SLC47A1 axis as a critical mechanism through which tumor cells resist ferroptosis, facilitating disease progression. These findings suggest that broad JAK-STAT inhibition may be counterproductive in cancer therapeutics; instead, therapeutic success depends on precise modulation and carefully timed interventions to preserve its T-cell-associated functions. This study may inspire future studies to explore specific factors that selectively modulate JAK-STAT activity in immune cells to achieve favorable therapeutic outcomes.

Strengths:

Significant therapeutics implications

Weaknesses:

Limited molecular mechanisms

Comments on revised version:

The authors have addressed my comments

# Author comment: Author response

**DOI:** 10.7554/eLife.110635.4.sa3

**Authors:** Jianbo Zhou, Heng Zhang, Hailin Tang, Lei Yu, Fu Peng

**Affiliations:** Sichuan University Chengdu China; Sichuan University Chengdu China; Sun Yat-sen University Cancer Center Guangzhou China; Chongqing Hospital of Traditional Chinese Medicine Chongqing China; Sichuan University Chengdu China

The following is the authors’ response to the previous reviews

**Public Reviews:**

**Reviewer #1 (Public review):**

Summary:

In their manuscript, Zhou and colleagues present a detailed look at how the JSP functions differently in the various cells of a breast tumor. The authors have effectively shown that the JSP acts as a double-edged sword, as it helps T cells fight cancer but also allows tumor cells to grow and avoid ferroptosis. These findings are important because they identify a useful biomarker to predict how TNBC patients might respond to PD-1 inhibitors.

Strengths:

This work is important because it provides a clear explanation for the conflicting roles of the JSP in the tumor environment. The evidence is solid, as it combines data from thousands of patients with single-cell analysis and lab experiments to confirm the role of STAT4 in cancer progression and immunity.

Comments on revised version:

The authors made a significant effort to improve the manuscript. My comments were sufficiently addressed.

We sincerely appreciate your careful review and positive feedback. We are glad to hear that you are satisfied with the revised manuscript and acknowledge the scientific value and solid evidence of our work. Thank you again for all your efforts and valuable suggestions.

**Reviewer #2 (Public review):**

Summary:

The JAK-STAT pathway (JSP) exhibits cell-type-specific functional heterogeneity in breast cancer. This study investigates the JSP in breast cancer and its response to anti-PD‑1 immunotherapy. JSP displays distinct cell‑type heterogeneity: it promotes malignant phenotypes and immunosuppression in tumor cells, while enhancing cytotoxicity and reducing exhaustion in T cells. Elevated JSP expression correlates with improved immunotherapy responses, especially in triple‑negative breast cancer. These findings highlight the paradoxical roles of JSP, indicating that broad inhibition may compromise anti‑tumor immunity.

Strengths:

The major strengths of this study include the comprehensive characterization JSP heterogeneity across epithelial, tumor, and T cells in breast cancer. The identification of JSP and STAT4 as predictive biomarkers for immunotherapy response, particularly in triple‑negative breast cancer, provides clinically relevant insights for patient stratification.

Weaknesses:

The corresponding content has been revised.

We sincerely thank you for your detailed review and valuable comments. We greatly appreciate your recognition of the cell-type-specific heterogeneity of the JAK-STAT pathway and the clinical value of JSP and STAT4 as predictive biomarkers for immunotherapy in triple-negative breast cancer. We have thoroughly revised the manuscript according to your previous suggestions, and all raised concerns have been fully addressed.

**Reviewer #3 (Public review):**

Summary:

This multi-omics study by Zhou et al elucidates the context-dependent roles of the Janus kinase-signal transducer and activator of transcription (JAK-STAT) pathway (JSP) across different cellular compartments in the breast cancer tumor microenvironment. While bulk JSP activity is associated with a favorable prognosis, single-cell analysis reveals a paradoxical landscape: high JSP in T cells drives anti-tumor cytotoxicity and reduces exhaustion, whereas high activity in tumor epithelial cells promotes malignancy and immunosuppression via the MIF-CD74 signaling axis. The JSP score (immune-related) serves as a robust predictive biomarker for response to anti-PD-1 immunotherapy, particularly in triple-negative breast cancer (TNBC). Furthermore, the study identifies the STAT4/SLC47A1 axis as a critical mechanism through which tumor cells resist ferroptosis, facilitating disease progression. These findings suggest that broad JAK-STAT inhibition may be counterproductive in cancer therapeutics; instead, therapeutic success depends on precise modulation and carefully timed interventions to preserve its T-cell-associated functions. This study may inspire future studies to explore specific factors that selectively modulate JAK-STAT activity in immune cells to achieve favorable therapeutic outcomes.

Strengths:

Significant therapeutics implications

Weaknesses:

Limited molecular mechanisms

Comments on revised version:

The authors have addressed my comments

Many thanks for your careful evaluation and valuable suggestions. We highly appreciate your affirmation of the therapeutic significance of this study. We have fully revised the manuscript to enrich the molecular mechanisms, and all your comments have been properly resolved.

**Recommendations for the authors:**

**Reviewer #2 (Recommendations for the authors):**

The most content has been revised.

Minor corrections:

(1) The icon about "prognosis" in graphic abstract is overly childish.

The graphical abstract has been redrawn. The inappropriate prognosis icon is deleted accordingly.

(2) Please double check the whole content to avoid typos. For instance, "2.2" and "2.3" have been repeated twice.

We appreciate your reminder. The duplicate numbering of 2.2 and 2.3 resulted from Word’s automatic heading feature. We have disabled this function and fixed all repeated section numbers. In addition, we have carefully checked the full text and corrected all typos.

(3) It will be more interesting if the oncogenic role of STAT4 could be verified via cell cloning assay.

We appreciate your thoughtful comment. Considering the limited revision time, we cannot add the cell cloning assay in the current version. Our present data sufficiently validate the oncogenic function of STAT4, and the main conclusions remain reliable.

**Reviewer #3 (Recommendations for the authors):**

I recommend publishing the revised manuscript in eLife.

We sincerely thank you for your positive evaluation and endorsement for the publication of our revised manuscript. We greatly appreciate your rigorous review and insightful comments that have substantially improved the quality and readability of this work.

We sincerely appreciate all reviewers and editors for your thorough reviewing work and thoughtful feedback. Your suggestions have helped us greatly improve this manuscript. Thank you very much.
